# A Novel Cryptic Clostridial Peptide That Kills Bacteria by a Cell Membrane Permeabilization Mechanism

**DOI:** 10.1128/spectrum.01657-22

**Published:** 2022-09-12

**Authors:** Monika Szadkowska, Michal Olewniczak, Anna Kloska, Elzbieta Jankowska, Malgorzata Kapusta, Bartosz Rybak, Dariusz Wyrzykowski, Wioletta Zmudzinska, Artur Gieldon, Aleksandra Kocot, Anna-Karina Kaczorowska, Lukasz Nierzwicki, Joanna Makowska, Tadeusz Kaczorowski, Magdalena Plotka

**Affiliations:** a Laboratory of Extremophiles Biology, Department of Microbiology, Faculty of Biology, University of Gdansk, Gdansk, Poland; b Department of Physical Chemistry, Gdansk University of Technology, Gdansk, Poland; c Department of Medical Biology and Genetics, Faculty of Biology, University of Gdansk, Gdansk, Poland; d Department of Biomedical Chemistry, Faculty of Chemistry, University of Gdansk, Gdansk, Poland; e Department of Plant Cytology and Embryology, Faculty of Biology, University of Gdansk, Gdansk, Poland; f Department of Environmental Toxicology, Faculty of Health Sciences with Institute of Maritime and Tropical Medicine, Medical University of Gdansk, Gdansk, Poland; g Department of General and Inorganic Chemistry, Faculty of Chemistry, University of Gdansk, Gdansk, Poland; h Laboratory of Biopolymer Structure, Intercollegiate Faculty of Biotechnology, University of Gdansk and Medical University of Gdansk, Gdansk, Poland; i Laboratory of Simulation of Polymers, Department of Theoretical Chemistry, Faculty of Chemistry, University of Gdansk, Gdansk, Poland; j Collection of Plasmids and Microorganisms, Faculty of Biology, University of Gdansk, Gdansk, Poland; Emory University School of Medicine

**Keywords:** antibacterial peptides, endolysins, membrane potential, molecular dynamics, toroidal pore model

## Abstract

This work reports detailed characteristics of the antimicrobial peptide Intestinalin (P30), which is derived from the LysC enzyme of Clostridium intestinale strain URNW. The peptide shows a broader antibacterial spectrum than the parental enzyme, showing potent antimicrobial activity against clinical strains of Gram-positive staphylococci and Gram-negative pathogens and causing between 3.04 ± 0.12 log kill for Pseudomonas aeruginosa PAO1 and 7.10 ± 0.05 log kill for multidrug-resistant Acinetobacter baumannii KPD 581 at a 5 μM concentration. Moreover, Intestinalin (P30) prevents biofilm formation and destroys 24-h and 72-h biofilms formed by Acinetobacter baumannii CRAB KPD 205 (reduction levels of 4.28 and 2.62 log CFU/mL, respectively). The activity of Intestinalin is combined with both no cytotoxicity and little hemolytic effect against mammalian cells. The nuclear magnetic resonance and molecular dynamics (MD) data show a high tendency of Intestinalin to interact with the bacterial phospholipid cell membrane. Although positively charged, Intestinalin resides in the membrane and aggregates into small oligomers. Negatively charged phospholipids stabilize peptide oligomers to form water- and ion-permeable pores, disrupting the integrity of bacterial cell membranes. Experimental data showed that Intestinalin interacts with negatively charged lipoteichoic acid (log*K* based on isothermal titration calorimetry, 7.45 ± 0.44), causes membrane depolarization, and affects membrane integrity by forming large pores, all of which result in loss of bacterial viability.

**IMPORTANCE** Antibiotic resistance is rising rapidly among pathogenic bacteria, becoming a global public health problem that threatens the effectiveness of therapies for many infectious diseases. In this respect, antimicrobial peptides appear to be an interesting alternative to combat bacterial pathogens. Here, we report the characteristics of an antimicrobial peptide (of 30 amino acids) derived from the clostridial LysC enzyme. The peptide showed killing activity against clinical strains of Gram-positive and Gram-negative pathogens. Experimental data and computational modeling showed that this peptide forms transmembrane pores, directly engaging the negatively charged phospholipids of the bacterial cell membrane. Consequently, dissipation of the electrochemical gradient across cell membranes affects many vital processes, such as ATP synthesis, motility, and transport of nutrients. This kind of dysfunction leads to the loss of bacterial viability. Our firm conviction is that the presented study will be a helpful resource in searching for novel antimicrobial peptides that could have the potential to replace conventional antibiotics.

## INTRODUCTION

The emergence and spread of antibiotic-resistant bacteria is a serious global public health problem affecting medical treatment effectiveness for many infectious diseases (1, 2). Many efforts are under way to combat pathogens more effectively (3). Antimicrobial peptides (AMPs) have been recognized as promising candidates for treating bacterial infections (4). The AMPs are ubiquitous and are found in both eukaryotes (e.g., fungi, plants, insects, and mammals) and prokaryotes (e.g., bacteria) (5). One AMP classification system is based on their source; other classification systems consider structural characteristics and activity (6). The AMPs are classified into α-helical, β-sheet, or peptides with an extended or random-coil structure (4). The primary target for AMPs is the bacterial cytoplasmic membrane, but some peptides can kill bacteria without affecting membrane integrity (7). Several studies have shown that an AMP’s secondary structure does not necessarily predict its mechanism of action (8). Therefore, characterization of the mode of action of novel AMPs is essential to accelerate their use as potential therapeutics.

The group of antimicrobial peptides generally omitted in the reviews are AMPs that are parts of larger proteins. These kinds of AMPs are called cryptic AMPs. So far, the phenomenon of hidden AMPs in the primary structures of proteins has been mainly associated with higher eukaryotes and is exemplified by such proteins as human apolipoprotein E (9) and collagen VI (10). The peptide ApoE, corresponding to residues 133 to 150 of human apolipoprotein E, possesses broad-spectrum antibacterial activity (9). Similarly, peptides derived from collagen VI exhibit significant antibacterial properties against Staphylococcus aureus, Escherichia coli, and Pseudomonas aeruginosa via cell membrane damage (10).

Recently, the phenomenon of cryptic AMPs was also explored by scientists working on enzymes with lytic function derived from bacteria or viruses of bacteria, called bacteriophages (11–13). Often lytic proteins are less effective against Gram-negative bacteria due to the protective outer membrane barrier that prevents them from reaching their target, peptidoglycan (14). However, some lysins have intrinsic antibacterial properties mediated via amphipathic helices or highly positively charged regions (15). Examples include PlyF307 bacteriophage lysin, which exhibits antibacterial activity against Gram-negative Acinetobacter baumannii (11), LysP53 lysin from A. baumannii phage 53, with bactericidal activity toward A. baumannii isolates (12), and LysAB2 endolysin, encoded by the A. baumannii phage ϕAB2 (13). Peptides derived from these proteins show high antibacterial potential. Still, efforts are focused mainly on finding new AMPs in lytic proteins of Gram-negative bacteria or their phages, neglecting lysins with a Gram-positive background.

Recently, based on the amino acid sequence similarity to endolysins of phages isolated from Icelandic hot springs (16–18), our group discovered and characterized in detail the LysC enzyme from Clostridium intestinale URNW, which is highly bactericidal against S. aureus ATCC 25923 (19). LysC belongs to the autolysin group of enzymes encoded by bacteria and mainly involved in peptidoglycan remodeling during bacterial cell growth and division (20). Autolysins are also considered potential anti-infective agents. Surprisingly, although from *in silico* analysis LysC was annotated as an amidase that cut the amide bond between the carbohydrate chain and stem peptide of peptidoglycan, variants with the disrupted activity of the catalytic center still showed bactericidal activity. Subsequently, detailed studies pointed out that this activity was mediated by the positively charged N-terminal region of the protein, whose function was unassigned.

Here, we examined the bactericidal activity of a natural peptide named Intestinalin (P30), comprising the first 30 residues of the LysC protein, against clinical isolates of Staphylococcus and a range of Gram-negative pathogens in comparison to the activity of the parental enzyme. The Intestinalin (P30) is the first cryptic peptide with antimicrobial activity derived from the enzyme from Gram-positive bacteria. We hypothesized that this natural peptide targets the bacterial cell membrane, triggering its depolarization and permeabilization. We used fluorescence microscopy, isothermal titration calorimetry, nuclear magnetic resonance (NMR) spectroscopy, and advanced molecular dynamics (MD) simulations to verify our assumptions.

## RESULTS

### Antibacterial spectrum of Intestinalin (P30).

Previously, we tested the LysC autolysin from Clostridium intestinale URNW (GenBank ERK30183.1) and the peptide Intestinalin (P30) derived from the LysC N-terminal region (amino acid sequence KNLLRRIRRKLRNKFSRSDVIKTPKIVEVN) against Staphylococcus aureus ATCC 25923 (19). The detailed antibacterial tests showed that the effect was dose dependent, and the Intestinalin peptide had higher antibacterial activity than the LysC enzyme. A peptide concentration as low as 5 μM caused complete eradication of 10^6^ S. aureus ATCC 25923 cells, whereas a similar effect could only be observed at a 21.5 μM concentration of LysC (see Fig. S1 in the supplemental material). To further explore the antibacterial potential of the Intestinalin peptide in comparison to that of the LysC protein, we studied the bactericidal activity of Intestinalin (5 μM) and LysC (21.5 μM) *in vitro* against the Gram-positive staphylococci and Gram-negative bacteria listed in Table 1. S. aureus ATCC 25923 served as a positive control in this test. LysC was active against all Gram-positive bacteria tested, Gram-negative Citrobacter braakii from the order *Enterobacterales*, and Gram-negative bacteria from the order *Pseudomonadales*, Acinetobacter baumannii and Pseudomonas aeruginosa. For Gram-positive bacteria, LysC showed the greatest activity against a clinical isolate of S. aureus KPD 425 (6.83 ± 0.10 CFU log reduction [mean ± standard deviation]) and the least against S. aureus KPD 740 (4.76 ± 0.27 CFU log reduction). Still, the overall antibacterial activity of LysC exceeded 4.5-log reduction in bacterial counts. Between A. baumannii and P. aeruginosa strains, the greatest activity was shown against A. baumannii KPD 581 (7.10 ± 0.05 logs), and the least activity was observed for P. aeruginosa PAO1 (2.03 ± 0.07 logs). The LysC enzyme was barely active against enterobacteria (other than Citrobacter braakii; 4.75 ± 0.04 CFU log reduction) and showed 0.74 ± 0.05 log reduction of Enterobacter cloacae KPD 297, 0.34 ± 0.15 log reduction of Escherichia coli KPD 217, and 0.40 ± 0.04 log reduction of Klebsiella pneumoniae KPD 298 cells.

**TABLE 1:** LysC and Intestinalin (P30) bactericidal activities against several Gram-positive and Gram-negative bacterial pathogens

Bacterial species	CFU log reduction^*a*^ (mean ± SD)		Origin and characteristics^*b*^
	LysC	P30	
S. aureus ATCC 25923 control	5.12 ± 0.14	5.09 ± 0.04	Clinical strain (intermediate resistance to CAZ and AMO)
S. aureus KPD 740	4.76 ± 0.27 (3.90 ± 0.15)	6.77 ± 0.04 (2.41 ± 0.06)	Clinical strain (E, CC, TE)
S. aureus KPD 425	6.83 ± 0.10 (7.18 ± 0.10)	6.83 ± 0.10 (5.35 ± 0.21)	Clinical strain (CIP, LVX, E, CC, TE, FOX)
S. epidermidis KPD 440	6.53 ± 0.08	6.53 ± 0.08	Clinical strain (GM, CIP, LVX, E, CC)
S. hominis KPD 910	5.42 ± 0.06	5.42 ± 0.06	Clinical strain (TE, VA)
*S. pettenkoferi* KPD 741	6.73 ± 0.06	6.73 ± 0.06	Clinical strain (CIP, LVX, E, CC)
A. baumannii CRAB KPD 205	6.93 ± 0.08	6.93 ± 0.08	Carbapenem-resistant clinical strain (PIP, TZP, CAZ, FEP, IMP, MEM, CIP, LVX, SXT)
A. baumannii MDR KPD 581	7.10 ± 0.05	7.10 ± 0.05	Multidrug-resistant clinical strain (AMP, AMC, TZP, CEP, CXM, FOX, CTX, CAZ, FEP, ETP, MEM, CIP, SXT, TOB)
A. baumannii KPD 735	6.97 ± 0.07	6.97 ± 0.07	Clinical strain (sensitive to all tested antibiotics)
A. baumannii RUH134	6.70 ± 0.08	6.70 ± 0.08	Reference strain of EU clone II (PIP, TE, GM, STX)
*C. braakii* KPD 218	4.75 ± 0.04	6.83 ± 0.08	Clinical strain (AMP, AMC, TZP, CEP, CXM, FOX, CTX, CAZ, FEP, CSL, ETP, SXT)
E. cloacae KPD 297	0.74 ± 0.05	7.31 ± 0.02	Clinical strain (AMP, AMC, TZP, CEP, CXM, FOX, CTX, CAZ, FEP, CSL, ETP, CIP, SXT)
E. coli KPD 217	0.34 ± 0.15	7.06 ± 0.15	Clinical strain (AMP, AMC, TZP, CEP, CXM, FOX, CTX, CAZ, FEP, CSL, SXT)
K. pneumoniae KPD 298	0.40 ± 0.04	5.28 ± 0.02	Clinical strain (AMP, AMC, TZP, CEP, CXM, FOX, CTX, CAZ, FEP, ETP, IMP, MEM, AKN, CIP, SXT, TOB)
P. aeruginosa PAO1	2.03 ± 0.07	3.04 ± 0.12	(AMP, PIP, TZP)
P. aeruginosa KPD 430	6.73 ± 0.10	6.73 ± 0.10	Clinical strain (PIP, TZP, CAZ, FEP, CIP, LVX, TCC, TOB)
P. aeruginosa CRPA KPD 431	4.69 ± 0.04	6.62 ± 0.12	Clinical strain (GM, PIP, TZP, CAZ, FEP, TCC, MEM)

a Values in parentheses for S. aureus strains KPD 740 and KPD 425 are results with 10^7^ cells rather than 10^6^.

b Patterns of antibiotic resistance were provided by the Department of Clinical Microbiology at University Clinical Centre, Gdansk, Poland, and are based on susceptibility tests performed according to the EUCAST method for antibiotic resistance. E, erythromycin; CC, clindamycin; TE, tetracycline; VA, vancomycin; PIP, piperacillin; TZP, piperacillin-tazobactam; CAZ, ceftazidime; FEP, cefepime; IMP, imipenem; MEM, meropenem; CIP, ciprofloxacin; LVX, levofloxacin; SXT, trimethoprim-sulfamethoxazole; AMP, ampicillin; AMO, amoxicillin; AMC, amoxicillin-clavulanic acid; AKN, amikacin; CEP, cephalothin; CXM, cefuroxime sodium; FOX, cefoxitin; CTX, cefotaxime; CSL, cefoperazone-sulbactam; ETP, ertapenem; TCC, ticarcillin-clavulanic acid; TOB, tobramycin; GM, gentamicin.

The Intestinalin peptide was active against all bacterial pathogens tested, with a specific reduction between 3.04 ± 0.12 logs for Pseudomonas aeruginosa PAO1 and 7.31 ± 0.02 logs for Enterobacter cloacae KPD 297 (Table 1). Intestinalin had a broader antibacterial spectrum encompassing E. cloacae KPD 297, E. coli KPD 217, and K. pneumoniae KPD 298, with activity exceeding 5.28-log reductions in bacterial counts, while the killing activity of LysC against these strains was in a range of 0.34- to 0.74-log decrease (Table 1). Therefore, we decided to investigate further the bactericidal potential of the Intestinalin (P30) peptide.

### Inhibition of biofilm formation.

**FIG 1:**
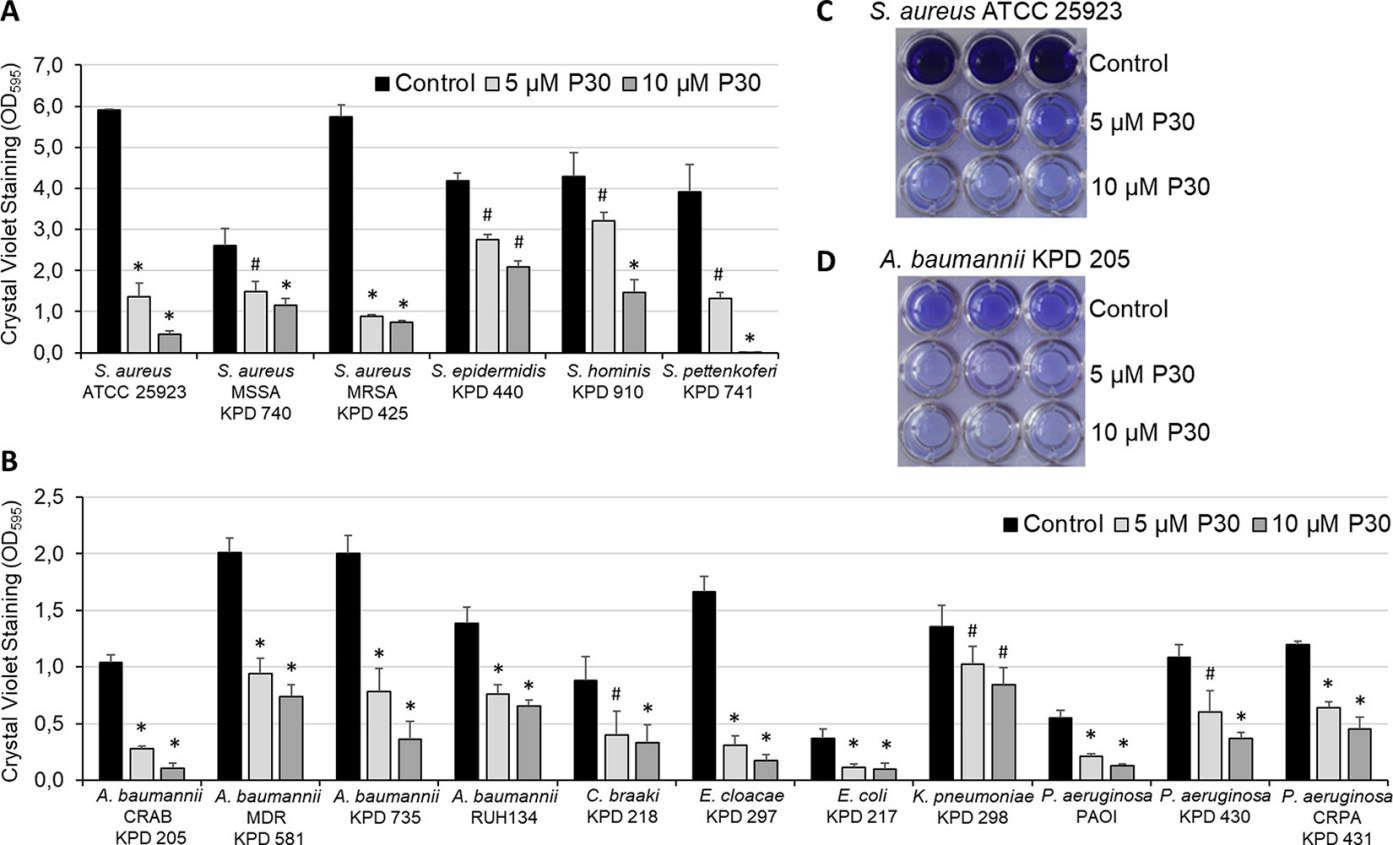
Inhibition of bacterial biofilm formation. (A and B) Effects of Intestinalin (P30) on biofilm formation of Gram-positive (A) and Gram-negative (B) bacteria incubated without (control) or with 5 μM or 10 μM peptide for 24 h at 37°C. Experiments were performed in triplicate; error bars indicate standard deviations. *, *P = *0.0001; #, *P ≤ *0.0002 (Student's *t* test). (C and D) Photograph showing the crystal violet-stained biofilm formed by S. aureus ATCC 25923 (C) and A. baumannii CRAB KPD 205 (D) in the absence (control) or presence of Intestinalin at concentrations of 5 μM and 10 μM.

The ability of Intestinalin (P30) to affect biofilm formation was evaluated by the crystal violet staining assay on six Gram-positive (Fig. 1A) and 11 Gram-negative bacterial strains (Fig. 1B). Biofilms of all tested strains were grown in triplicates in a 96-well microtiter plate for 24 h at 37°C in the presence of 5 μM or 10 μM Intestinalin. The planktonic cells were then removed, and the adhered biomass was stained with crystal violet and dissolved in 33% acetic acid, followed by absorbance determination. Examples of acetic acid-dissolved biofilms without (control) or in the presence of Intestinalin at concentrations of 5 μM and 10 μM are shown for S. aureus ATCC 25923 (Fig. 1C) and A. baumannii CRAB KPD 205 (Fig. 1D). In all cases, significant inhibition of biofilm formation was recorded (Fig. 1 and Table 2). For Intestinalin at a concentration of 10 μM, biofilm formation was significantly reduced by 99.7% for S. pettenkoferi KPD 741 in the group of Gram-positive bacteria and by 89.7% for two Gram-negative bacteria, A. baumannii CRAB KPD 205 and E. cloacae KPD 297 (*P = *0.0001). Lower inhibition, but still statistically significant, was observed in the case of 5 μM Intestinalin. The lowest inhibitions of biofilm formation were 25.4% for S. hominis KPD 910 (Gram-positive bacteria) and 24.3% for K. pneumoniae KPD 298 (Gram-negative bacteria) (*P ≤ *0.0002).

**TABLE 2:** Inhibition of bacterial biofilm formation by Intestinalin (P30) in relation to untreated controls

Bacterial strain	% inhibition of biofilm formation (mean ± SD)	
	5 μM	10 μM
Gram-positive bacteria		
S. aureus ATCC 25923	76.8 ± 5.6	92.3 ± 1.2
S. aureus MSSA KPD 740	42.9 ± 9.2	56.2 ± 6.7
S. aureus MRSA KPD 425	84.7 ± 0.7	87.1 ± 0.7
S. epidermidis KPD 440	34.4 ± 3.3	50.3 ± 3.4
S. hominis KPD 910	25.4 ± 4.8	66.1 ± 7.3
*S. pettenkoferi* KPD 741	66.1 ± 3.8	99.7 ± 0.3
Gram-negative bacteria		
A. baumannii CRAB KPD 205	73.3 ± 2.5	89.7 ± 3.9
A. baumannii MDR 581	53.2 ± 6.8	63.3 ± 5.1
A. baumannii KPD 735	60.9 ± 10.0	82.0 ± 7.8
A. baumannii RUH134	45.1 ± 5.9	53.1 ± 8.1
*C. braaki* KPD 218	54.3 ± 23.6	62.0 ± 17.8
E. cloacae KPD 297	81.5 ± 4.8	89.7 ± 3.1
E. coli KPD 217	70.2 ± 8.5	73.6 ± 14.0
K. pneumoniae KPD 298	24.3 ± 11.5	38.1 ± 11.2
P. aeruginosa PAOI	60.9 ± 3.5	77.3 ± 3.6
P. aeruginosa KPD 430	44.5 ± 17.8	66.0 ± 5.3
P. aeruginosa CRPA KPD 431	46.3 ± 4.3	62.2 ± 8.7

### MIC.

The MIC values were determined for Intestinalin (P30) against a panel of Gram-positive and Gram-negative bacteria listed in Table 3. Intestinalin (P30) showed MIC values ranging from 7.8 to >124 μM. The highest MICs (> 124 μM) were observed for S. aureus KPD 740, S. aureus KPD 425, E. cloacae KPD 297, E. coli KPD 217, and K. pneumoniae KPD 298. The lowest MICs (7.8 μM) were against three of four A. baumannii strains tested, as well as for S. hominis KPD 910 and *S. pettenkoferi* KPD 741. Interestingly, Intestinalin (P30) had greater antimicrobial activity against bacteria from the order *Pseudomonadales*, A. baumannii and P. aeruginosa, than against S. aureus and bacteria from the *Enterobacteriaceae* family. These results were not observed in antibacterial tests performed in a hypotonic environment (20 mM HEPES, pH 7.4), where most bacteria were similarly susceptible to the activity of the peptide.

**TABLE 3:** MICs of Intestinalin (P30) against Gram-positive and Gram-negative bacterial strains

Bacterial strain	P30 MIC	
	μg/mL	μM
Gram-positive bacteria		
S. aureus ATCC 25923	455	124
S. aureus KPD 740	>455	>124
S. aureus KPD 425	>455	>124
S. epidermidis KPD 440	455	124
S. hominis KPD 910	28.5	7.8
*S. pettenkoferi* KPD 741	28.5	7.8
Gram-negative bacteria		
A. baumannii CRAB KPD 205	28.5	7.8
A. baumannii MDR KPD 581	28.5	7.8
A. baumannii KPD 735	28.5	7.8
A. baumannii RUH134	114	31
*C. braakii* KPD 218	114	31
E. cloacae KPD 297	>455	>124
E. coli KPD 217	>455	>124
K. pneumoniae KPD 298	>455	>124
P. aeruginosa PAO1	57	15.5
P. aeruginosa KPD 430	228	62
P. aeruginosa CRPA KPD 431	57	15.5

### Cytotoxicity assays.

**FIG 2:**
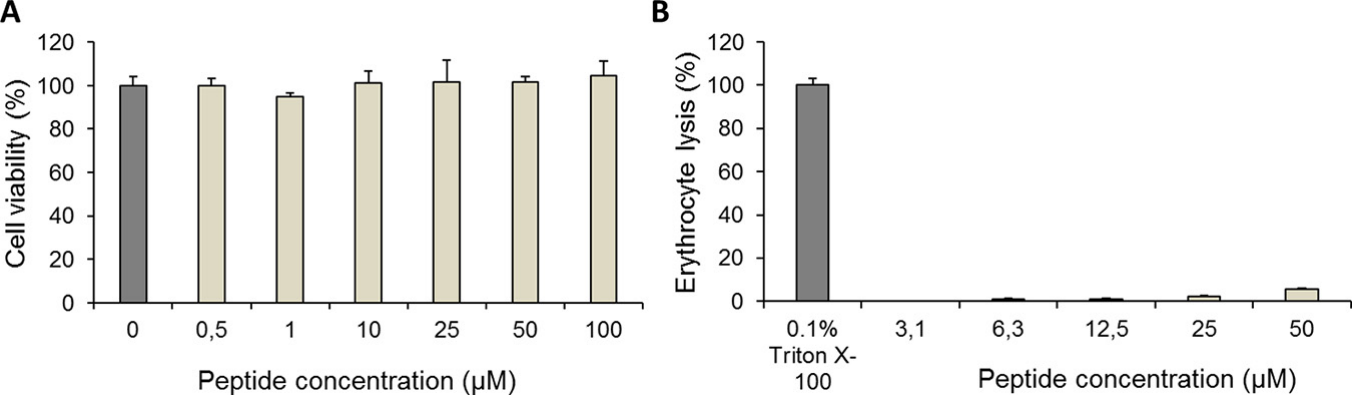
Viability of human keratinocyte (HaCaT) cells and insignificant lysis of erythrocytes upon Intestinalin (P30) exposure. (A) Cell viability was assessed by the MTT assay and compared with control cells grown in a peptide-free medium. Samples were incubated for 72 h at 37°C. Values represent means of at least two independent experiments. (B) The erythrocyte lysis assay checked the hemolytic effect of Intestinalin. Erythrocytes were collected from fresh sheep blood and incubated with different concentrations (3.1 to 50 μM) of Intestinalin (P30) for 1 h at 37°C. A 0.1% solution of Triton X-100 served as the positive control. Negative control results with 10 mM phosphate-buffered saline (pH 7.3) were subtracted from the sample measurements. Experiments were performed in triplicate; error bars indicate standard deviations.

Often, strong hemolytic activity and mammalian cytotoxicity hamper the clinical use of antibacterial peptides. To exclude this possibility, we analyzed the toxicity of Intestinalin (P30) against aneuploid immortalized human keratinocyte (HaCaT) cells by the 3-(4,5-dimethylthiazol-2-yl)-2,5-diphenyl tetrazolium bromide (MTT) assay. The results showed that Intestinalin was not toxic to HaCaT cells at concentrations up to 100 μM (Fig. 2A). Moreover, Intestinalin (P30) showed only 5.7% ± 0.4% hemolytic activity in an erythrocyte lysis assay at 50 μM and <2.2% activity at concentrations of ≤25 μM (Fig. 2B). Erythrocytes treated with 0.1% Triton X-100 served as a positive control (100% erythrocyte lysis).

### Intestinalin (P10) targets the bacterial cell membrane.

Because Intestinalin (P30) exerts high bactericidal activity against bacterial pathogens and shows no toxicity to human keratinocytes, it has high therapeutic potential. However, Intestinalin is just a short peptide derived from a larger protein. What is its mechanism of action? Previous, circular dichroism (CD) studies showed that in the presence of a cell membrane-mimicking detergent, Intestinalin formed an α-helix (19). Therefore, we hypothesized that Intestinalin targets bacterial cell membranes similarly to other polycationic α-helical peptides (21).

To investigate the relationship between function and conformation of Intestinalin (P30), we intended to perform NMR spectroscopy to reveal the structure of a 30-amino-acid peptide in the presence of SDS, a detergent known to mimic bacterial cell membranes. However, we could not make the correct signal assignment for the whole peptide, due to a very high overlapping signal. Therefore, proton signals of the Intestinalin (P10)_SDS peptide (LysC_2–12_; sequence, KNLLRRIRRK) were assigned based on total correlation spectroscopy (TOCSY) spectra, and the sequential analysis was confirmed by rotating-frame Overhauser effect spectroscopy (ROESY). We identified many signals in the ROESY spectrum which are characteristic of a helical structure, like *d*NN (i, i + 1) or *d*aN (i, i + 3) (see Fig. S2). Moreover, we identified many side chain proton interactions between (i, i + 3) and (i, i + 4) residues (see Table S1). The chemical shifts of the proton resonances for Intestinalin (P10)_SDS are listed in Table S2. Our study clearly showed that P10 peptide adopts an α-helical-like conformation in the presence of negatively charged lipids.

**FIG 3:**
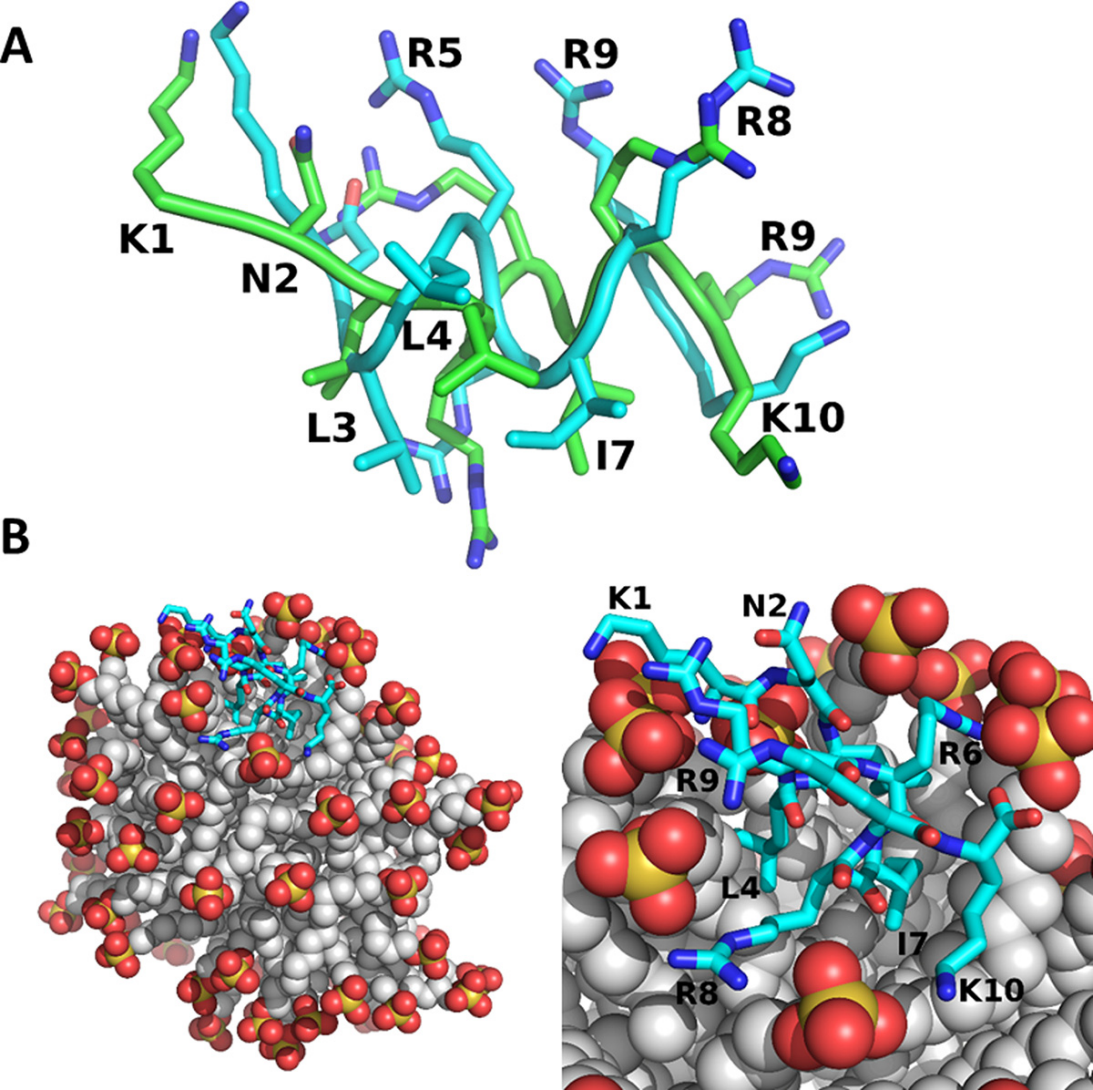
Molecular dynamics simulation of Intestinalin (P10) with SDS. (A) The best-fitting conformation of the Intestinalin (P10) peptide to the experimental one was estimated by NMR studies (RMSD, 1.05 Å). (B) Visualization of SDS molecular dynamics simulation results with the Intestinalin (P10) peptide. Sulfate groups are marked as yellow and red balls. The gray balls represent a hydrophobic chain of the SDS molecule. The peptide backbone is colored blue.

Subsequently, to shed light on the mechanism of Intestinalin (P10) peptide interaction with the lipid bilayer, a 1-μs-long run of conventional molecular dynamics (MD) simulations was used to examine the binding of Intestinalin (P10) to micelles composed of SDS. Based on the NMR and CD findings, we concluded that Intestinalin's (P10) structure is helical. We noted that the peptide remained in close contact with the micelle during the whole simulation length. We performed a clustering procedure to examine the possible conformational states of Intestinalin (P10) bound to the SDS micelle. We found that the peptide populated two forms: α-helical and random coil during 35% and 65% of the trajectory, respectively (see Fig. S3A). Using NMR spectroscopy we obtained a conformation of a micelle-bound Intestinalin (P10), and we compared its structure with the ensemble obtained with MD trajectory. The best-fitting solution was found for the helical structures with a root mean square deviation (RMSD) of about 1.05 Å (Fig. 3A) and an average RMSD for the helical conformation of 2.73 ± 0.37 Å.

Intestinalin (P10) was bound to the SDS micelle throughout the whole simulation length, so we examined the interactions that stabilize the P10-micelle complex. We found a large number of salt bridges formed by the positively charged residues of the peptide with negatively charged sulfate groups of the SDS (Fig. 3B), with the most stable interactions formed by R5, R6, R9, and K10 (Fig. 3B; see also Fig. S3B). This interaction is critical in the first step of antibacterial action of the peptide, as a large number of salt bridges determine the stability of the bilayer-peptide interaction, which prevents diffusion of the peptide.

### Intestinalin (P30) oligomer structure and activity in the cell membrane.

Our initial SDS micelle study showed that shorter Intestinalin (P10) strongly binds to the negatively charged lipids. We explored interactions of the intact, 30-amino-acid-long peptide with the bacterial cell membrane using Martini coarse-grained molecular dynamics simulations. Intestinalin (P30) is a part of the lytic protein derived from the Gram-positive bacterium Clostridium intestinale URNW. Therefore, in our test, we used the membrane model of Gram-positive S. aureus, a causative agent of many nosocomial infections. The simulation system contained 16 α-helical Intestinalin (P30) peptides embedded in a bilayer composed of 1-palmitoyl-2-oleoyl-*sn*-glycero-3-phosphoglycerol (POPG) and cardiolipin in a 4:1 ratio, which mimics the cell membrane of S. aureus (22). The simulations were performed in five replicas yielding a total of 50 μs of sampling, providing reliable statistics for analyzing Intestinalin (P30) interactions with the cell membrane. During our simulations, all Intestinalin (P30) peptides remained in close contact with the cell membrane, albeit in a dynamic equilibrium between membrane-spanning and surface-associated peptides (see Fig. S4).

**FIG 4:**
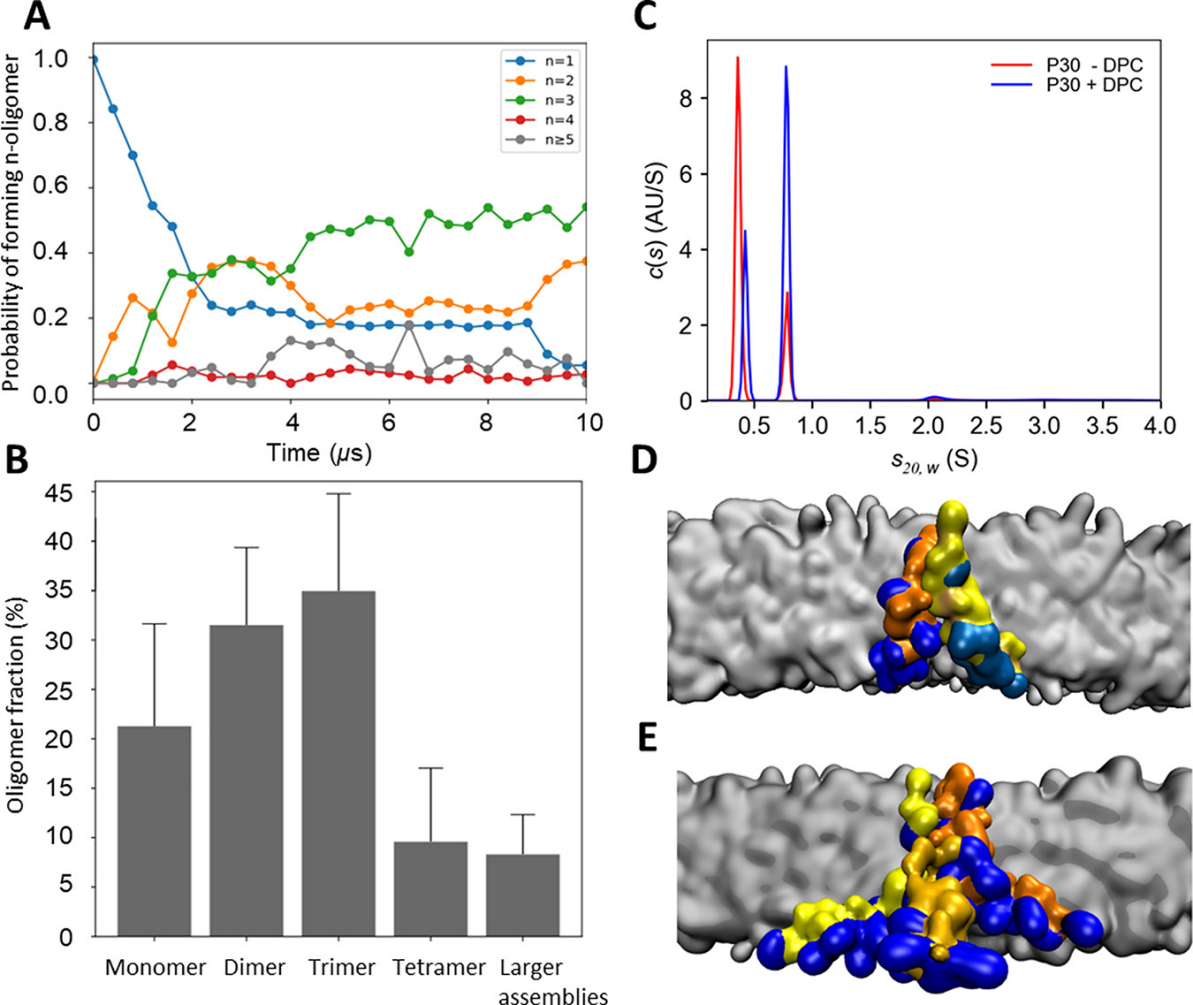
The oligomeric state of Intestinalin (P30). (A) The change of oligomer fraction in time for a single coarse-grained simulation replica. After initial equilibration (~3 μs), the fractions of oligomers remained at the same level. The fraction of large oligomers (*n* > 4) represents the collision complexes of small oligomers. (B) Oligomer fractions of Intestinalin (P30) in the cell membrane, computed with coarse-grained molecular dynamics (CG MD). (C) The AUC sedimentation coefficient distribution profiles of Intestinalin (P30) in the absence (red line) and presence (blue line) of DPC detergent. (D and E) Snapshot from the CG MD of the Intestinalin (P30) dimer (D) and trimer (E) embedded in the cell membrane. The blue color indicates positively charged residues of each monomer, which are colored orange, light, and dark yellow.

To evaluate the orientation preference of Intestinalin (P30) with respect to the cell membrane, a probability distribution of the angle between the main axis of the peptide helical domain and a membrane normal was computed. The fraction of membrane-spanning peptides was 2 times greater than the surface-associated orientation, resulting in ~1 kcal/mol preference for the transmembrane orientation of Intestinalin (P30). Notably, our results showed that Intestinalin (P30) tends to form multimers in the cell membrane (Fig. 4A). The size of peptide oligomers was examined, assuming that Intestinalin (P30) monomers form oligomeric structures when a minimal distance between them is <0.7 nm. As presented in Fig. 4B, Intestinalin (P30) formed dimers, trimers (predominantly), and to a lower extent tetramers in the cell membrane.

Next, analytical ultracentrifugation (AUC) was used to experimentally verify the degree of oligomerization of the Intestinalin (P30) peptide in the presence or absence of *n*-dodecylphosphocholine (DPC) detergent that, similarly to SDS, mimics bacterial cell membranes. As a result of AUC, two main peaks were found in both cases, and they accounted for ~94% of the signal (Fig. 4C). In the case of Intestinalin (P30) without DPC, the dominance of the first peak was noticeable and corresponded to the monomer (with a sedimentation coefficient of 0.36 *s*_20,_*_w_*) and was responsible for ~72% of the signal. The accompanying second peak (0.78 *s*_20,_*_w_*) represented only 22% of the signal. The addition of detergent changed the intensity of the peaks. The first one (0.42 *s*_20,_*_w_*) was responsible for only 27% of the signal. The dominant role was taken over by the second (0.78 *s*_20,_*_w_*), corresponding to Intestinalin (P30) in oligomeric form, which accounted for over 66% of the signal. The analysis based on the model with a bimodal frictional ratio, *f*/*f*_0_, allowed for the independent calculation of frictional ratio coefficients for each population (23). The results indicated that a monomer of a globular shape formed the first peak (frictional ratio,1.46), and the second peak most likely represented a trimer of elongated or irregular shape (frictional ratio, 2.80).

In the oligomeric state, Intestinalin (P30) monomers faced each other with positively charged regions (Fig. 4D and E), forming a channel of polar residues, which further supports the notion that Intestinalin (P30) induces disruption of the bacterial membrane, and as a consequence, the collapse of cell membrane chemiosmotic potential.

### Mechanism of Intestinalin (P30) antimicrobial activity based on all-atom MD.

**FIG 5:**
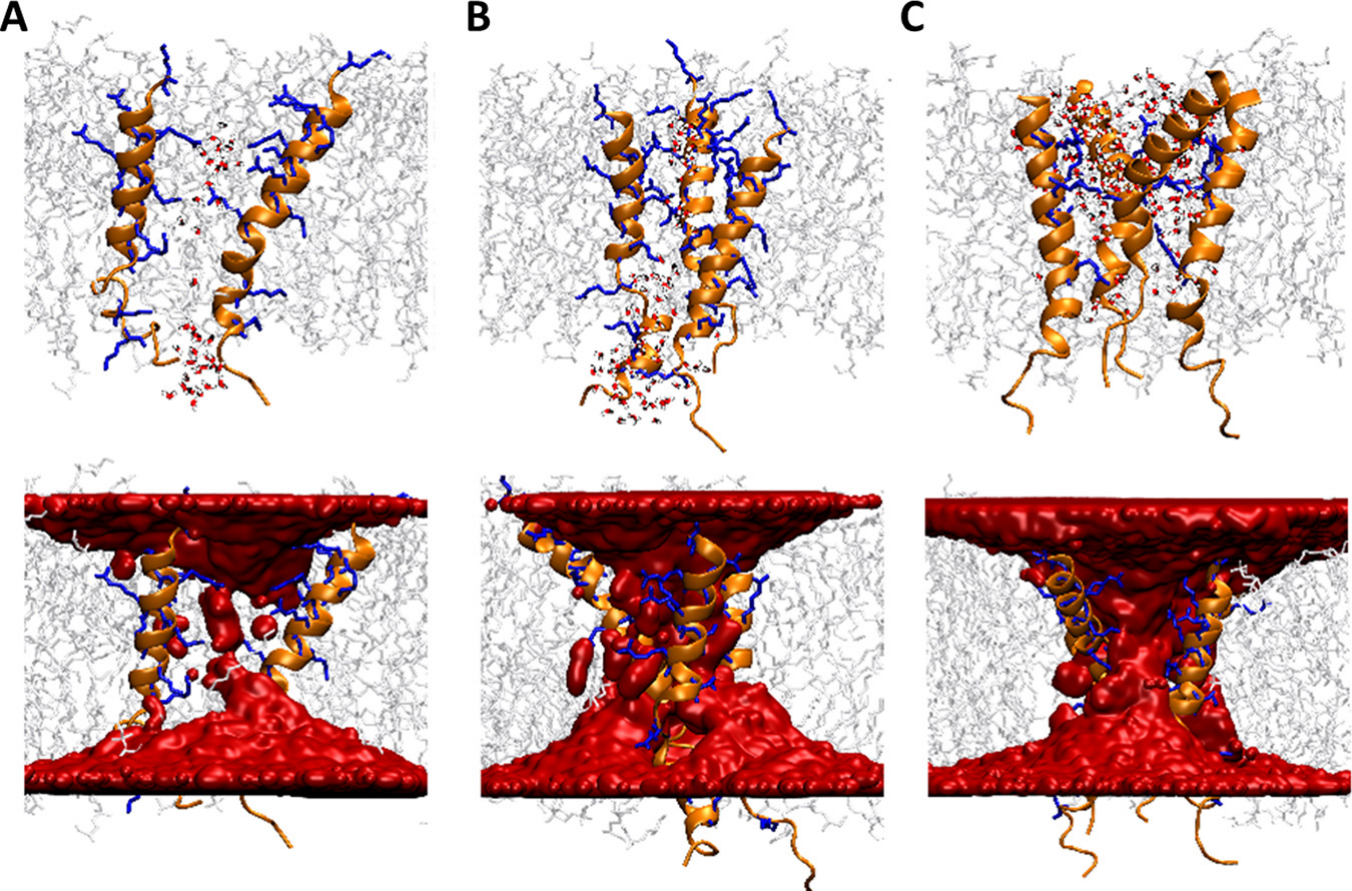
Molecular representation of Intestinalin (P30)-mediated water pores (top) and water density within the cell membrane (bottom). (A) Intestinalin (P30) dimer; (B) trimer; (C) tetramer.

We performed for each possible oligomeric state (identified with coarse-grained MD) 500-ns-long all-atom MD simulations to investigate the impact of Intestinalin (P30) oligomers on the cell membrane of S. aureus. As a result, we found that Intestinalin (P30) peptides remained in close contact facing each other with polar residues (Fig. 5A to C), similar to the coarse-grained MD results.

Our MD analyses indicated that many water molecules interacted with polar residues of the transmembrane segments of Intestinalin (P30) oligomers. We computed the average spatial distribution of water in proximity to each oligomer to quantify the amount of water that enters the cell membrane due to its interactions with Intestinalin (P30). As shown in Fig. 5, a continuous water density was observed through the entire membrane, demonstrating that Intestinalin (P30) oligomers form channels that enable water to permeate freely through the cell membrane.

**FIG 6:**
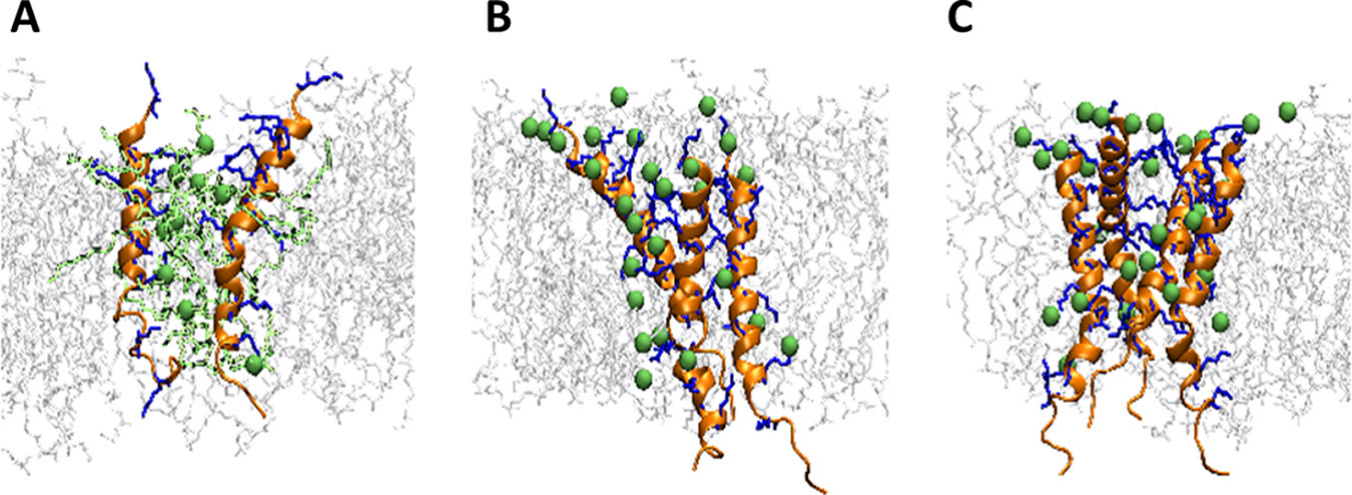
Molecular representations of Intestinalin (P30) interactions with lipids within the water pore. (A) Intestinalin (P30) dimer; (B) trimer; (C) tetramer. The phosphate groups of lipids interacting with Intestinalin (P30) within the water pore are depicted with green spheres. In the case of the trimer and tetramer, the lipid tails were removed for clarity.

Finally, the interaction strength between Intestinalin (P30) peptides and other system components (peptide oligomerization partners, cell membrane phospholipids, and water) was examined to clarify the origins of Intestinalin (P30) oligomers' stability. Notably, the favorable interaction energy of the polar lipid headgroups and peptide monomers was 1 magnitude higher than the repulsion between Intestinalin (P30) monomers (see Table S3), suggesting the lipids’ major role in stabilizing Intestinalin (P30) oligomers. Surprisingly, for each oligomer, we observed a significant increase in favorable interactions between the lipid headgroups and peptide oligomers in the initial part of the simulation. To explore this phenomenon, we visually examined the orientation of the lipids in proximity to each Intestinalin (P30) oligomer. Surprisingly, during the initial 30 ns, the lipid headgroups of both POPG and cardiolipins in proximity of Intestinalin (P30) oligomer moved from their original surface orientation to locate between the positively charged residues of Intestinalin (P30) within the water channel (Fig. 6A to C). We concluded that these newly formed interactions between Intestinalin (P30) cationic residues and negatively charged lipid headgroups are critical for stabilizing oligomers as they outweigh the electrostatic repulsion between Intestinalin (P30) monomers (as shown in Table S3).

### Intestinalin (P30) binds LTA and causes bacterial cell membrane depolarization and its permeabilization.

**FIG 7:**
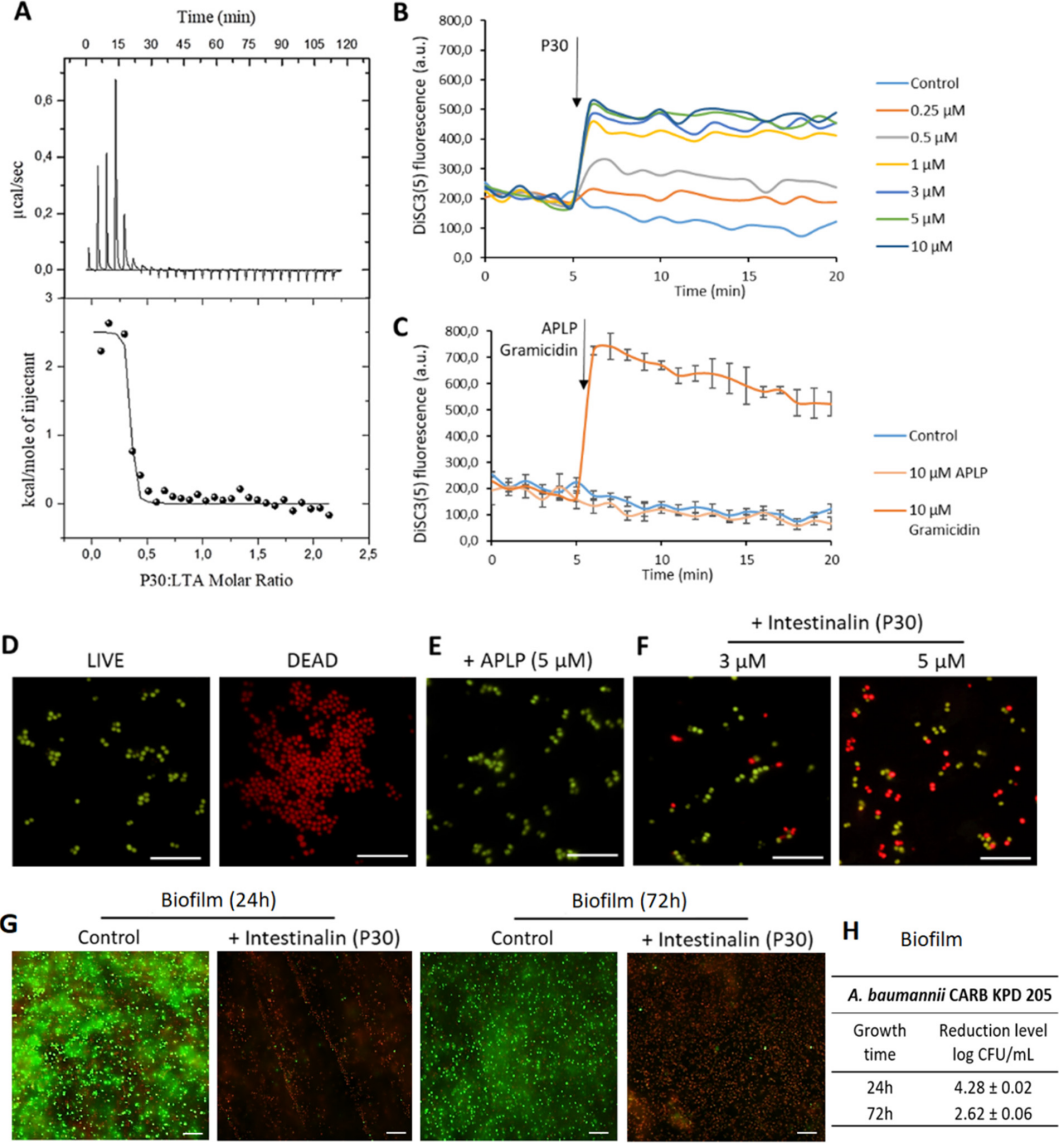
Mechanism of action of Intestinalin (P30). (A) Interaction between Intestinalin (P30) and LTA. Calorimetric titration isotherm of the binding interaction between LTA and Intestinalin (P30) in 20 mM HEPES (pH 7.4) at 25°C. (B) The membrane potential-sensitive dye DiSC_3_(5) was incubated with bacteria until a stable baseline was formed. The time point of the P30 addition is highlighted with an arrow. Fluorescence increase upon adding peptide was measured at an excitation wavelength of 652 nm and emission wavelength of 672 nm as a function of time. Error bars were omitted for clarity. (C) Positive and negative controls are shown in separate graphs. The time point of APLP and gramicidin addition is highlighted with an arrow. Experiments were performed in triplicate; error bars indicate standard deviations. (D to F) Results of the bacterial viability test visualized with fluorescence microscopy. (D) Metabolically active S. aureus ATCC 25923 cells are stained green (LIVE), and dead cells with membrane damage are stained red (DEAD). (E) S. aureus cells (10^8^) after incubation in the presence of 5 μM APLP (negative control). (F) Cells after incubation with 3 μM and 5 μM Intestinalin (P30). Bar, 10 μm. (G) Biofilm of A. baumannii CRAB KPD 205 in a LabTek chamber slide system (Thermo Fisher Scientific) after 24 h and 72 h of growth in the control experiment and after incubation with Intestinalin (P30) at twice the MIC (15.6 μM) for 3 h. (H) Reduction levels of A. baumannii CARB KPD 205 biofilms formed in a 24-well microtiter plate in the control and after incubation with Intestinalin (P30) at twice the MIC (15.6 μM) for 3 h.

The MD simulations clearly showed that Intestinalin (P30) forms channels in the bacterial cell membrane. First, to verify theoretical predictions, we employed isothermal titration calorimetry (ITC) to analyze Intestinalin (P30) binding to negatively charged components of bacterial membranes. The electrostatic interactions between cationic AMP and negatively charged lipoteichoic acids (LTAs) in Gram-positive bacteria can modulate the adherence of peptides to the bacterial surface (24). The thermodynamic parameters of the Intestinalin (P30)-LTA interactions were obtained directly from ITC measurements by fitting isotherms (using nonlinear least-squares procedures) to a model that assumed one set of binding sites (Fig. 7A). This model gave the best fit of the calculated data to experimental ones. Then, the standard thermodynamic relationships for the free energy of binding (Δ*G*_ITC_, = −R*T*lnK_ITC_ = Δ*H*_ITC_ − TΔ*S*_ITC_) were used to calculate the free energy of binding (Δ*G*_ITC_, −10.17 ± 0.60 kcal mol^−1^) and the entropy change (TΔ*S*_ITC_, 12.68 ± 0.61 kcal mol^−1^). Calorimetric data revealed that Intestinalin (P30) forms a relatively stable complex with LTA (log*K*_ITC_ = 7.45 ± 0.44). Furthermore, under experimental conditions (pH 7.4), the formation of the P30-LTA complex is an entropy-driven process (|*T*Δ*S*_ITC_| > |Δ*H*_ITC_|). The positive value of Δ*H*_ITC_, 2.51 ± 0.10 kcal mol^−1^, indicated that hydrophobic interactions involved in binding events play a pivotal role in stabilizing the resulting P30-LTA complex (25).

Subsequently, we used the voltage-sensitive dye 3,3′-dipropylthiadicarbodocyanine iodide [DiSC_3_(5)] to analyze cell membrane potential upon addition of Intestinalin (P30) to an S. aureus ATCC 25923 cell suspension. The carbocyanine dye DiSC_3_(5) accumulates on a polarized cell membrane, resulting in fluorescence self-quenching. Upon cell membrane depolarization, the dye is released and fluorescence increases. The addition of Intestinalin induced a significant increase of DiSC_3_(5) fluorescence in S. aureus (Fig. 7B). The increase was rapid at concentrations between 1 μM and 10 μM, while the peptide at concentrations of 0.25 μM and 0.5 μM showed a smaller effect (Fig. 7B). The addition of 10 μM gramicidin (a mixture of gramicidin A, B, C, and D) was used as a positive control, and 10 μM APLP peptide (which does not have antibacterial activity) served as a negative control (Fig. 7C).

Next, fluorescence microscopy based on the LIVE/DEAD bacterial viability test was used to analyze potential cell membrane permeabilization caused by Intestinalin (P30) (Fig. 7D to G). Green fluorescent SYTO 9 and red fluorescent propidium iodide (PI) dyes, both of which intercalate into the DNA, were utilized in this assay. PI penetrates only cells with larger pores or holes in the bacterial cell membranes and is generally excluded from viable cells, while SYTO 9 enters live and dead bacterial cells. When both dyes are present, PI exhibits a stronger affinity for nucleic acids than SYTO 9, and PI displaces SYTO 9. Therefore, live bacteria with an intact cell membrane stain green, and dead bacteria with extensive cell membrane damage stain red (Fig. 7D). Adding APLP peptide (1.9 kDa) to 10^8^ S. aureus ATCC 25923 cells did not cause any harm to treated bacteria (Fig. 7E). Incubation of cells in the presence of Intestinalin (P30) (3 μM) resulted in the appearance of dead cells. Their number increased with a higher concentration of peptide (5 μM) added to the sample, indicating that cell membrane integrity was critically damaged due to the extensive formation of pores that allowed PI to enter the cells (Fig. 7F). The impact of Intestinalin (P30) on biofilm formed by A. baumannii CRAB KPD 205 showed the same effect, and incubation with peptide resulted in the appearance of dead cells (Fig. 7G). Moreover, Intestinalin (P30) activity on biofilm was confirmed by reductions of 4.28 and 2.62 log CFU/mL after 24 and 72 h of biofilm development, respectively (Fig. 7H).

In addition, a kinetic kill and depolarization assay was employed to detect membrane depolarization and permeabilization simultaneously (see Fig. S5). S. aureus ATCC 25923 cells were incubated with Intestinalin (P30), LL-37 (positive control), or with APLP peptide (negative control) in 20 mM HEPES (pH 7.4) containing 1 μM DiSC_3_(5) and 5 μg/mL PI at 37°C in 96-well black microtiter plates. Fluorescence was measured throughout the assay, and aliquots were taken after 60-min incubation to determine bacterial viability by CFU counts. The addition of Intestinalin (P30) induced a rapid, significant increase of both DiSC_3_(5) (see Fig. S5A) and PI fluorescence (see Fig. S5B) at concentrations of ≥3 μM. At a 3 μM concentration of the peptide, 5.00 ± 0.06 log CFU/mL reduction in viable S. aureus cells was observed (see Fig. S5C and D). At higher concentrations of the peptide (>3 μM), high fluorescence levels of DiSC_3_(5) and PI correlated well with the complete eradication of bacterial cells. Altogether, the data showed that membrane depolarization and permeabilization cooccurred after peptide addition, and these findings correlate with the peptide’s bactericidal activity and support the conclusions presented in the manuscript.

As expected, the APLP peptide did not show antibacterial activity, and levels of DiSC_3_(5) and PI fluorescence were comparable to that in the control without adding the peptide. In the positive control (10 μM LL-37), high levels of DiSC_3_(5) and PI fluorescence were detected, and the peptide at this concentration caused complete eradication of S. aureus cells.

## DISCUSSION

Intestinalin (P30) is an interesting example of the antimicrobial peptide being part of a larger protein. Our results show that the panel of naturally occurring antibacterial peptides with high bactericidal potential is wider than previously thought.

Notably, Intestinalin (P30) showed potent activity against Gram-negative bacteria, reducing the viability of the tested strains in antibacterial tests by at least 3 logs (3.04 ± 0.12 log reduction of P. aeruginosa PAO1) and showing an average of 7-log reduction of A. baumannii cells. Therefore, the antimicrobial activity of Intestinalin (P30) is much higher than, for example, P307 peptide (part of PlyF307 lysin), which caused an average 2.7-log decrease in the bacterial count of 19 A. baumannii strains tested and showed MICs for four A. baumannii strains of ≥221 μM (11). Moreover, Intestinalin (P30) was much more effective against Staphylococcus aureus in antibacterial tests. These bacteria were only moderately sensitive to P307, with a ~1.3-log average decrease (11). Another peptide, P87, derived from Pae87 endolysin from Pseudomonas aeruginosa phage JG004, was also barely active against Gram-positive bacteria (26). Intestinalin has a broader antibacterial spectrum than P307 and P87 peptides, and most importantly, a lower dose can be applied (13, 16). Subsequently, Intestinalin (P30) can efficiently interfere with bacterial biofilm formation, causing biofilm biomass reduction ranging from 24.3% for K. pneumoniae to 99.7% for *S. pettenkoferi*. This finding is significant, as approximately 80% of human infections are caused by bacterial biofilms formed on biotic or abiotic surfaces (27, 28). The anti-biofilm formation activity of Intestinalin is similar to that of previously described antimicrobial peptides, such as Esculentin-1a (29), Nisin A, lacticin Q, Nukacin ISK-1 (30), and RN3(5-17P22-36) (31), which disrupt or degrade the membrane potentials of biofilm-embedded cells.

Another important feature of Intestinalin (P30) is the lack of toxicity to mammalian cells, as potential toxic side effects of AMPs have hampered their clinical development (32). Selectivity of Intestinalin toward bacterial cells can be explained by the high affinity of the positively charged peptide to negatively charged phospholipid components of the bacterial membrane. As mammalian cytoplasmic membranes are mainly composed of zwitterionic phospholipids and cholesterol, the lack of such an affinity to neutral lipids explains the low toxicity of Intestinalin against mammalian cells (33). Intestinalin shows a small hemolytic effect, which can be correlated with the moderate (33%) hydrophobicity of the peptide (predicted by CAMP_R3_ [available at http://www.bicnirrh.res.in/antimicrobial]) (32, 34). The higher hydrophobicity of AMPs is associated with a stronger tendency of these peptides to aggregate in an aqueous solution. This aggregation enhances the hemolytic activity of such peptides because, in that form, they can penetrate deeper into the hydrophobic core of the red blood cell membrane (34). At the same time, strong self-association of peptides in solution prevents their access to the bacterial cell membrane (34). That is in line with our results showing that Intestinalin is (i) predominantly monomeric in the aqueous environment and forms oligomers just in the presence of cell membranes, (ii) does not cause lysis of sheep erythrocytes when present at concentrations up to 25 μM, and (iii) has high bactericidal activity against Gram-positive staphylococci and Gram-negative bacteria. All these data suggest that Intestinalin is a potent antimicrobial peptide, which is a good starting point for further research, including *in vivo* models of bacterial infections.

Our subsequent work was directed toward clarifying the detailed mechanism of Intestinalin (P30) activity. Interaction with cell wall components is the first step in attracting the AMPs to the bacterial surface (24). ITC measurements showed that Intestinalin (P30) binds LTAs with high affinity. It has been postulated that LTAs form a polyanionic ladder that helps cationic AMPs traverse the cell membrane of Gram-positive bacteria (35). In Gram-negatives, the role of LTAs is overtaken by lipopolysaccharide and outer membrane proteins that attract AMPs to the bacterial surface (21). That is probably the case with Intestinalin (P30), as the peptide is highly active against Gram-negative pathogens.

AMPs usually disrupt the cytoplasmic membrane, but the polypeptide antibiotic gramicidin, which destroys Gram-positive bacteria, forms ion channels too small to be permeable for the fluorescent dye PI (36). As revealed by experiments using DiSC_3_(5) and PI dyes, Intestinalin (P30) caused membrane depolarization and permeabilization, forming pores large enough for PI to pass through. These data were supported by coarse-grained and all-atom MD simulations showing that Intestinalin (P30) is a membrane-spanning peptide and forms oligomers in the bacterial cell membrane. The mechanisms of action of transmembrane pore-forming peptides that cause membrane depolarization are divided into two main categories: the toroidal pore and the barrel stave models (32). Because self-associated Intestinalin (P30) forms transmembrane pores that directly engage the negatively charged phospholipids of the bacterial cell membrane, the toroidal pore model best describes the antibacterial activity of the peptide. Dissipation of the electrochemical gradient of protons across the cell membrane affects many processes, such as ATP synthesis, motility by flagellar rotation, and transport of nutrients. This kind of dysfunction leads to the loss of bacterial viability.

Insufficient understanding of AMPs’ activities and the relationships between their structure and bactericidal function has slowed progress in developing new AMPs. Therefore, the results presented here provide valuable insights into the molecular mechanism of action of a novel antimicrobial peptide, Intestinalin (P30). That knowledge may be used for the future design of AMPs or modular lytic enzymes with desired properties. Importantly, our results point out that novel AMPs can be found in the sequences of already-known antimicrobial proteins; as shown in this Intestinalin (P30) example, such AMPs could potentially have higher antimicrobial activities than their parental enzymes.

## MATERIALS AND METHODS

### Bacterial strains, plasmids, and culture conditions.

Staphylococcus aureus ATCC 25923 was purchased from the American Type Culture Collection and Pseudomonas aeruginosa PAO1 (DSM 22644) was from Deutsche Sammlung von Mikroorganismen und Zellkulturen GmbH, Germany. Acinetobacter baumannii RUH134 was obtained from Leiden University Medical Center, The Netherlands. Clinical strains of Staphylococcus aureus KPD 740, S. aureus KPD 425, S. epidermidis KPD 440, S. hominis KPD 910, *S. pettenkoferi* KPD 741, A. baumannii CARB KPD 205, A. baumannii MDR KPD 581, A. baumannii MDR KPD 735, Citrobacter braakii KPD 218, Enterobacter cloacae KPD 297, Escherichia coli KPD 217, Klebsiella pneumoniae KPD 298, Pseudomonas aeruginosa KPD 430, and P. aeruginosa CRPA KPD 431 were provided by the Collection of Plasmids and Microorganisms (KPD) at the University of Gdansk, Poland, with patterns of antibiotic resistance available for each strain. Staphylococcal strains were cultured in tryptic soy broth (TSB; Graso Biotech, Starogard Gdanski, Poland) or a solid TSB medium containing 1.5% (wt/vol) agar at 37°C. All other bacterial strains were grown in Luria-Bertani (LB) broth or LB agar at 37°C. pET-LysC plasmid for overproduction of LysC enzyme was prepared as previously described (19). For protein overproduction in E. coli BL21(DE3), LB was supplemented with 100 μg/mL of ampicillin.

### Protein purification and peptide synthesis.

The His-tagged recombinant LysC enzyme was purified using cobalt-based immobilized metal affinity chromatography (19). Fractions containing pure protein were dialyzed against 20 mM HEPES (pH 7.4) for activity testing or against 20 mM HEPES (pH 7.4) and 50% glycerol for storage at −80°C. Intestinalin (P30) was synthesized as previously described (19).

### Antibacterial assays.

Bacterial substrates were grown in TSB or LB medium at 37°C to exponential phase (optical density at 660 nm [OD_600_] of ~0.45 to 0.5). Cells were harvested at 4,000 × *g* for 15 min at 20°C, washed with 20 mM HEPES (pH 7.4), and resuspended in the same buffer. For the antimicrobial activity assays, LysC at a final concentration of 22.5 μM (500 μg/mL) or Intestinalin (P30) at a concentration of 5 μM (20 μg/mL) were added to approximately 10^6^ bacterial cells (10-fold dilution) in 20 mM HEPES (pH 7.4) in a final volume of 300 μL. The equivalent volume of 20 mM HEPES (pH 7.4) was added to the negative control instead of tested antibacterial agents. After 1.5 h of incubation at 37°C, reaction mixtures were serially diluted in 20 mM HEPES (pH 7.4) and plated. Simultaneously, 5-μL drops were plated onto TSB or LB agar plates to perform spot dilution assays. CFU were counted after overnight incubation at 37°C. The antibacterial activity was presented in logarithmic units [log_10_(*N*_0_/*N_i_*), where *N*_0_ is the number of untreated cells (in the negative control) and *N_i_* is the number of treated cells counted after incubation]. All experiments were performed in triplicate.

### MICs.

The MICs were determined by a broth microdilution assay in 96-well microtiter plates, using Mueller-Hinton (MH) broth with log-phase bacterial strains (listed in Table 2), following the Clinical and Laboratory Standards Institute (CLSI) guidelines (37). Briefly, bacteria were grown overnight at 37°C in MH broth. The next day, the number of mid-log-phase bacterial cells was quickly adjusted to 10^6^ CFU/mL in the same medium and cells were transferred into 96-well microtiter plates (90 μL/well). Twofold serial dilutions of Intestinalin (P30) were prepared in H_2_O in polypropylene test tubes and added to the microtiter plate in a volume of 10 μL per well in triplicate to achieve final concentrations of the peptide ranging from 124 to 3.9 μM. Bacteria were allowed to grow at 37°C for 24 h. The CLSI defines the MIC as the lowest peptide concentration that completely inhibits visible bacterial growth (38).

### Biofilm assays.

Overnight bacterial cultures were adjusted to a 0.5 McFarland standard in fresh TSBg (TSB supplemented with 0.25% glucose) corresponding to 1 × 10^8^ CFU/mL and an OD_625_ of ~0.08 to 0.1. Dilutions (100×) were made in TSBg, and 200-μL aliquots from these suspensions were used to inoculate the required number of wells of Nunclon Delta polystyrene 96-well microtiter plates (Thermo Fisher Scientific, Waltham, MA, USA). Cells were incubated under static conditions for 24 h at 37°C without or in the presence of 5 μM or 10 μM Intestinalin in a 1-μL volume. Following the incubation, wells were washed twice with phosphate-buffered saline (PBS; 137 mM NaCl, 2.7 mM KCl, 10 mM Na_2_HPO_4_, and 2 mM KH_2_PO_4_; pH 7.4) and 200 μL of 0.1% crystal violet dye was added to each well. After 15 min of incubation, the excess dye was removed, wells were washed twice with sterile water, and the dye attached to each well was solubilized with 200 μL of 33% acetic acid. The absorbance at 595 nm was then measured with a Sunrise absorbance microplate reader (Tecan, Männedorf, Switzerland). Data obtained from three independent biological replicates were analyzed by a two-tailed, unpaired Student's *t* test using GraphPad Prism 5.0 software (https://www.graphpad.com/quickcalcs/ttest1.cfm). *P* values of <0.05 were considered significant. The biofilms of A. baumannii CARB KPD 205 were prepared in 24-well microtiter plates (Costar, Corning, NY). After 24 h and 72 h of growth, the biofilms were treated with Intestinalin (P30) at twice the MIC (15.6 μM) for 3 h at 37°C. Sterile saline (0.85% NaCl) was used as a control. The biofilms were harvested with sterile swabs, vortexed, diluted, and spread plated onto LB agar plates. The results are expressed as reduction levels in log CFU per milliliter.

### Cell viability assay.

Cell viability was assessed in a 3-(4,5-dimethylthiazol-2-yl)-2,5-diphenyltetrazolium bromide (MTT) reduction assay. Human keratinocyte cell line HaCaT (CLS Cell Lines Service GmbH, Eppelheim, Germany) was grown in Dulbecco's modified Eagle medium (Gibco, Thermo Fisher Scientific) supplemented with 10% heat-inactivated fetal bovine serum (Gibco, Thermo Fisher Scientific) and 1× antibiotic-antimycotic solution (Gibco, Thermo Fisher Scientific), at 37°C in a humidified atmosphere containing 5% (vol/vol) carbon dioxide. Dulbecco's PBS (DPBS; LifeTechnologies, Carlsbad, CA, USA) was used to wash cells before treatment with 0.25% trypsin–EDTA solution (Gibco, Thermo Fisher Scientific) to detach cells. The MTT working solution used in the cell viability assay was prepared by dissolving MTT (Sigma-Aldrich, St. Louis, MO, USA) to the concentration of 1 mg/mL in RPMI 1640 medium without phenol red (Sigma-Aldrich). HaCaT cells were plated at 10^4^ cells per well in 96-well plates and incubated overnight under standard culture conditions to allow attachment. The growth medium was substituted with a medium supplemented with tested peptide solutions at concentrations of 0.5 to 100 μM. Cells were exposed to the tested agent for 24 or 72 h. Next, the medium was removed, and cells were incubated with MTT working solution at 37°C for 1 h. Formazan precipitate was dissolved in dimethyl sulfoxide (DMSO; Sigma-Aldrich), and the absorbance was read at 570 nm (including blanks) in a microplate reader (VICTOR^3^ multilabel plate reader; PerkinElmer, Waltham, MA, USA). Cell viability was calculated relative to viability of untreated control cells.

### Erythrocyte lysis assay.

Erythrocytes were harvested from 5.0 mL of fresh sheep blood (BioMaxima S.A., Gdansk, Poland) by centrifugation at 1,300 × *g* for 10 min at 20°C and washed three times with 10 mM PBS, pH 7.3 (Merck Life Science). Next, 100 μL of erythrocyte suspension diluted with 10 mM PBS (final concentration, approximately 2%) was added to each well of a 96-well microtiter plate and incubated for 1 h at 37°C in the presence of 100 μL of 2-fold serial dilutions of the peptide in PBS at concentrations ranging from 0.8 to 200 μM. Values for 0% and 100% lysis were determined by incubation of the erythrocytes with 10 mM PBS (pH 7.3) and 0.1% (vol/vol) Triton X-100, respectively. After incubation, plates were centrifuged at 1,300 × *g* for 10 min at 20°C, and the supernatant was transferred to a new plate. The absorbance was measured spectrophotometrically at 540 nm using a Sunrise absorbance microplate reader (Tecan). All experiments were performed in triplicate.

### Preparation of the sample for the NMR experiment.

SDS-d25 was purchased from Sigma-Aldrich. The sample in SDS micelles was prepared as described elsewhere (39) at a concentration of 3 mM Intestinalin (P10)_SDS in 0.7 mL of water solution (90% H_2_O–10% D_2_O) containing over 35 mg of SDS-d25. The concentration of SDS-d25 exceeded the critical micelle concentration of SDS.

### ^1^H NMR spectroscopy.

The NMR spectra of Intestinalin (P10) at 25°C were measured on a Varian 500-MHz spectrometer (Varian Medical Systems, Crawley, UK). The following two-dimensional ^1^H NMR spectra were recorded: double quantum-filtered correlation spectroscopy, TOCSY (80 ms), and ROESY and nuclear Overhauser effect spectroscopy (100 ms and 300 ms). The spectra were processed using the Varian software and analyzed with the Sparky program (40).

### MD simulations of Intestinalin (P10)_SDS complex.

The calculation protocol which was used in this study to determine the conformational ensemble of the Intestinalin (P10) peptide in complex with the SDS micelle (as for the NMR conditions) has been described, validated and used with good results previously (41–44). The Intestinalin (P10) was placed on the surface of the SDS micelle, composed of 70 SDS molecules placed in an 8.3-by-8.3-by-8.3-Å rectangular box filled with 18,216 water molecules and 114 K^+^ and 50 Cl^−^ ions to provide physiological ionic strength. In this case, the hydrogen mass repartitioning approach (45) was used, which enabled us to use the 4-fs time step of integration. Obtained trajectories were clustered with the gmx_cluster tool using a 0.3 nm RMSD cutoff for the heavy atoms of the Intestinalin (P10) peptide. Minimal distances between SDS sulfate groups and P10 side chains were computed using gmx mindist (46).

### Coarse-grained study of the P30 oligomerization process.

The coarse-grained systems were built using the CHARMM-GUI server (47) and simulated using Gromacs 2020 (46). The Martini model was used for both the protein (version 2.2) (48) and the rest of the system (version 2.0) (49). Each of five copies of the simulated system was composed of 16 evenly distributed P30 monomers embedded in the lipid bilayer, which was composed of POPG and cardiolipin in a 4:1 ratio (2,400 lipid molecules in total), which mimics the membrane composition of S. aureus (22). The bilayer systems were solvated with 48,224 Martini polarizable water particles (50) with the addition of 3,664 Na^+^ and 544 Cl^−^ ions to obtain the 150 mM salt concentration. A constant temperature of 303.15 K was maintained with the v-rescale thermostat (51), while constant pressure was set to 1 bar using the semi-isotropic Parrinello-Rahman barostat (52) with a relaxation time of 12 ps and the compressibility factor of 3·10^−4^/10^5^ Pa. Nonbonded interactions were evaluated using the potential shift method with a cutoff radius equal to 1.1 nm. Each copy of the system was simulated for 10 μs with the time step of 20 fs.

The change of the oligomer fractions in time was computed with a homemade python script utilizing the MDtraj library (53). As for oligomers, we quantified the peptide aggregates within which the minimal distance between any of the peptides was shorter than 7 Å. The oligomerization was computed for the trajectory frames with 10-ns intervals. The data points shown in Fig. 4A were obtained by averaging the fractions of each oligomer over the trajectory bins of 400 ns. The final fractions of the oligomers (Fig. 4B) were obtained as averages over five trajectories, discarding the initial 3 μs from each trajectory as an equilibration of the system.

The final radial distribution function (RDF) of P30 in the membrane plane was obtained with gmx_rdf and averaged over all peptides and all generated trajectories. The distribution of the tilt angle of P30 peptides was obtained by measuring the angle between the principal axis of the P30 helical part (obtained with the gmx_principal tool) and the normal vector of the membrane.

### Analytical ultracentrifugation.

Sedimentation velocity measurements were carried out in a ProteomeLab XL-I analytical ultracentrifuge (Beckman-Coulter, Indianapolis, IN, USA) equipped with An-50 8-hole analytical rotor and 12-mm path length and double-sector charcoal-Epon cells. The peptide samples were spun in the buffer containing 10 mM sodium bicarbonate, 5 mM sodium phosphate (pH 6.8), and 100 mM NaCl in the presence or absence of 20 mM DPC detergent. Both samples, without or with 20 mM DPC, had similar absorbances (0.64 and 0.65, respectively; 12-mm optical path, 230 nm). Cells containing 400 μL of sample and 410 μL of buffer were centrifuged at 50,000 rpm and monitored by UV absorbance at 230 nm at 20°C, using the continuous scan mode and radial spacing of 0.003 cm. Data were analyzed using the “Continuous c(s) distribution” model of the SEDFIT program (54), with a confidence level (*F*-ratio) specified at 0.68. Solvent densities (1.00416 g/cm^3^ without DPC and 1.00475 g/cm^3^ with DPC) and viscosities (1.006 mPa s without DPC and 1.034 mPa s with DPC) were measured at 20°C using a DMA 5000 densitometer (Anton Paar GmbH, Graz, Austria) and a Lovis 2000 M rolling-ball viscometer, respectively. The peptide’s particle-specific volume and extinction coefficient were calculated using SEDNTERP software (55). The results were plotted using the GUSSI graphical program (56).

### All-atom MD simulation setup.

All simulated systems were built using CHARMM-GUI server (47) and were performed using Gromacs 2020 (46) with the Plumed 2.6 plugin (57) and applying the CHARMM36m force field (58). The TIP3P model was used for water molecules, and the numbers of K^+^ and Cl^−^ ions were adjusted to match the 150 mM concentration. The Nose-Hoover algorithm (59) with a coupling constant of 1 ps was used to keep the temperature at 300.15 K, while the Parrinello-Rahman algorithm (60) with a coupling time of 5 ps was used to maintain the pressure of 10^5^ Pa. Electrostatic interactions were calculated with the particle mesh Ewald method (61) using a cutoff radius equal to 1.2 nm (with the switching function applied from the distance of 1 nm) and Fourier grid spacing of 0.12 nm. Van der Waals interactions were calculated with the Lennard-Jones potential with a cutoff radius of 1 nm. To integrate the equations of motion, the leap-frog Verlet algorithm was used with the timestep of 2 fs. VMD (62) and the GROmaρs toolset (63) were used for the molecular images.

### All-atom MD simulations of P30 oligomers.

Simulated systems were composed of P30 oligomers (dimer, trimer, tetramer, or pentamer) embedded in a phospholipid bilayer composed of POPG and tetralinoleoyl cardiolipin (TLCL2) in a 4:1 ratio, which mimicked the membrane of S. aureus (see Table S4 in the supplemental material). Starting structures of *N*-mers were obtained by repeated rotation of the fully helical *k*th P30 monomer by *2k*π/*N* about a selected center of geometry. After the standard CHARMM-GUI minimization procedure, systems were equilibrated for 150 ns and then simulated for 500 ns. Obtained trajectories were clustered with the gmx_cluster tool using a 0.25 nm RMSD cutoff for the backbone of the helical part of P30 in each case; the resulting output contained only one centroid. The water flux through those structures was then visualized with the gmx_spatial tool using values of the beta parameter of >0.05. In calculating the energy of interaction in oligomers, helical parts of the P30 were chosen as residues 1 to 23, while lipid head groups corresponded to the phosphoglycerol parts of POPG and TLCL.

### Isothermal titration calorimetry.

All ITC experiments were performed at 25°C using the AutoITC isothermal titration calorimeter (MicroCal Inc.). The measuring devices and experimental setup details were described previously (64). The reagents, P30 and LTA (Sigma-Aldrich catalog number L2515-5MG), were dissolved directly in 20 mM HEPES, pH 7.4. The experiment consisted of injecting 10.02 μL (29 injections, 2 μL for the first injection only) of 0.5 mM buffered solution of P30 into the reaction cell, which initially contained 0.05 mM buffered solution of LTA. A background titration, consisting of an identical titrant solution but with the buffer solution in the reaction cell only, was removed from each experimental titration. The LTA solution was injected at 4-min intervals. Each injection lasted 20 s. The stirrer speed was kept constant at 300 rpm. The CaCl_2_-EDTA titration was performed to check the apparatus, and the results (stoichiometry, *K*, and Δ*H*) were compared with those obtained for the same samples (a test kit) at Malvern Instruments Ltd. (Malvern, UK).

### Determination of membrane potential.

S. aureus ATCC 25923 cells were grown at 37°C in TSB medium to exponential phase (OD_600_ of ~0.45 to 0.5). Cells were harvested at 4,000 × *g* for 15 min at 20°C, washed with 10 mM PBS (pH 7.3), and 10× diluted in the same buffer supplemented with 1% DMSO to obtain ~10^6^ cells. The cell suspension was mixed with 1 μM DiSC_3_(5) dissolved in DMSO, and 200 μL was added to the wells of a black 96-well polystyrene microtiter plate (OptiPlate; PerkinElmer) (36). The plate was preincubated at 37°C for 25 min in the dark. Subsequently, fluorescence was monitored (excitation 652 nm, emission 672 nm) at 1-min intervals using a multimode plate reader (EnSpire 2300; PerkinElmer). When the readings were stabilized, the plate was ejected, and Intestinalin (P30) peptide was added in triplicate to a final concentration between 0.25 μM and 10 μM. The fluorescence was measured again for 15 min at 1-min intervals. A 10 μM concentration of gramicidin (Sigma-Aldrich) and 10 μM APLP peptide served as positive and negative controls, respectively. The APLP peptide (sequence, RVGGLEEERESVGPLRE; APLP peptide has no antibacterial activity and is a part of human amyloid-like protein 2, which has been implicated in the pathogenesis of Alzheimer's disease) was provided by the Department of Biomedical Chemistry, Faculty of Chemistry, University of Gdansk, Gdansk, Poland.

### Fluorescence microscopy.

S. aureus ATCC 25923 cells were grown in TSB medium at 37°C to the exponential phase (OD_600_ of ~0.45 to 0.5). Bacteria were centrifuged (7,000 × *g*, 7 min, 20°C), washed, and resuspended in 20 mM HEPES (pH 7.4). Intestinalin (P30) at concentrations of 3 μM and 5 μM was added to 3 × 10^8^ bacterial cells in 20 mM HEPES (pH 7.4) in a final volume of 500 μL, and the reaction mixtures were kept for 1 h at 37°C with gentle mixing every 15 min. For the positive control (DEAD cells), bacteria were incubated with 70% isopropyl alcohol instead of 20 mM HEPES (pH 7.4). The APLP peptide (5 μM) was used as a negative control (LIVE cells). After incubation, cells were washed, suspended in 500 μL of 20 mM HEPES (pH 7.4), and stained with the LIVE/DEAD BacLight bacterial viability kit (Invitrogen Molecular Probes, Eugene, OR) according to the protocol issued by the manufacturer. Briefly, 0.5 mL of bacterial suspension was stained with 1.5 μL of a 1:1 mixture of green fluorescent SYTO 9 (3.34 mM) and red fluorescent PI (20 mM) and incubated at room temperature (approximately 20°C), in the dark, for 15 min. Stained bacteria (3 μL) were immobilized on a 1% agarose pad, placed on a glass microscope slide, and examined with a Nikon Eclipse E800 epifluorescence microscope. Images were collected and processed with Lucia Laboratory Imaging Software (Laboratory Imaging s.r.o.). The fluorescence from both live and dead bacteria was viewed simultaneously with a Nikon blue excitation B-2A (long-pass emission) filter. Additionally, the biofilm of A. baumannii CARB KPD 205 was prepared in an experimental system according to methods described by Kocot and Olszewska (65). Briefly, 1 mL of A. baumannii in the exponential phase of growth was placed in the LabTek chamber slide system (Thermo Fisher Scientific). After 24 h and 72 h of growth, the biofilms were treated with Intestinalin (P30) at twice the MIC (15.6 μM), and sterile saline (0.85% NaCl) was used as a control. The biofilms in the LabTek system were stained with a LIVE/DEAD BacLight bacterial viability kit and examined as described above.

### Kinetic kill and depolarization assay.

Cells of mid-log-phase S. aureus ATCC 25923, suspended at 1 × 10^6^ CFU/mL in 20 mM HEPES (pH 7.4), were incubated with 1 μM DiSC_3_(5) and 5 μg/mL PI in the presence of 1% DMSO. The suspension was mixed by short vortexing, and 200 μL was added to the wells of a black 96-well plate (Optiplate; PerkinElmer). The plate was preincubated at 37°C for 25 min in the dark. Subsequently, fluorescence was monitored [excitation at 652 nm and emission at 672 nm for DiSC_3_(5), and excitation at 535 nm and emission at 617 nm for PI] at 1-min intervals using a VarioskanFlash 4.00.53 multimode microplate reader (Thermo Fisher Scientific). When the readings were stabilized, the plate was ejected. During a 10-min break, the Intestinalin (P30) peptide was added in triplicate to a final concentration between 0.25 μM and 10 μM. A 10 μM concentration of LL-37 antimicrobial peptide (kindly provided by the Department of Biomedical Chemistry, Faculty of Chemistry, University of Gdansk, Gdansk, Poland) and 10 μM APLP peptide served as positive and negative controls, respectively. The fluorescence was measured again for 15 min at 1-min intervals. At the end of incubation (≈60 min), aliquots were withdrawn from each well, serially diluted, and plated on TSB agar to perform spot dilution assays and allow CFU determination.
