# Supplementary material for: A Novel Cryptic Clostridial Peptide That Kills Bacteria by a Cell Membrane Permeabilization Mechanism
Source: Microbiol Spectr. 2022 Sep 12;10(5):e01657-22. doi: 10.1128/spectrum.01657-22 (PMC9602519; doi:10.1128/spectrum.01657-22)
Supplement: Supplemental file 1 — Fig. S1 and S2, Tables S1 and S2, Fig. S3 and S4, Tables S3 and S4, and Fig. S5. Download spectrum.01657-22-s0001.pdf, PDF file, 1.1 MB [file spectrum.01657-22-s0001.pdf]

A novel cryptic clostridial peptide that kills bacteria by cell membrane permeabilization mechanism

Monika Szadkowska,[a]* Michal Olewniczak,[b]* Anna Kloska,[c] Elzbieta Jankowska,[d] Malgorzata Kapusta,[e] Bartosz Rybak,[f] Dariusz Wyrzykowski,[g] Wioletta Zmudzinska,[h] Artur Gieldon,[i] Aleksandra Kocot,[a] Anna-Karina Kaczorowska,[j] Lukasz Nierzwicki,[b] Joanna Makowska,[g] Tadeusz Kaczorowski,[a] Magdalena Plotka[a]#

[a]Laboratory of Extremophiles Biology, Department of Microbiology, Faculty of Biology, University of Gdansk, Gdansk, Poland
[b]Department of Physical Chemistry, Gdansk University of Technology, Gdansk, Poland
[c]Department of Medical Biology and Genetics, Faculty of Biology, University of Gdansk, Gdansk, Poland
[d]Department of Biomedical Chemistry, Faculty of Chemistry, University of Gdansk, Gdansk, Poland
[e]Department of Plant Cytology and Embryology, Faculty of Biology, University of Gdansk, Gdansk, Poland
[f]Department of Environmental Toxicology, Faculty of Health Sciences with Institute of Maritime and Tropical Medicine, Medical University of Gdansk, Gdansk, Poland
[g]Department of General and Inorganic Chemistry, Faculty of Chemistry, University of Gdansk, Gdansk, Poland
[h]Laboratory of Biopolymer Structure, Intercollegiate Faculty of Biotechnology, University of Gdansk and Medical University of Gdansk, Gdansk, Poland
[i]Laboratory of Simulation of Polymers, Department of Theoretical Chemistry, Faculty of Chemistry, University of Gdansk, Gdansk, Poland
[j]Collection of Plasmids and Microorganisms, Faculty of Biology, University of Gdansk, Gdansk, Poland

Table of contents:

Figure S1. Spot dilution assays show the dose-dependent activity of LysC bacteriolytic enzyme and Intestinalin (P30) peptide against *S. aureus* ATCC 25923.

Figure S2. The ROE effects correspond to the interproton distances of Intestinalin (P10) at T = 25°C. The bars' thickness reflects the ROE correlation's strength as a strong, medium, or weak.

Table S1. Atoms of residues at separation |i – j| > 1, between which the ROE peaks were found at 25°C for Intestinalin (P10) peptide.

Table S2. ^{1}H chemical shifts (ppm) of Pep10_SDS in SDS-d25/D_2O at 25°C.

Figure S3. Molecular dynamics simulation of SDS with Intestinalin (P10).

Figure S4. MD simulation snapshots showing the position of P30 peptides in the membrane at the beginning of the simulation (t = 0 μs) and after the time evolution of the system (at t = 1 μs, 5 μs, or 10 μs).

Table S3. Electrostatic interactions for Intestinalin (P30).

Table S4. Simulated systems details.

Figure S5. Membrane depolarization ($DiSC_3(5)$), permeabilization (PI), and killing of *S. aureus* ATCC 25923 cells by Intestinalin (P30).

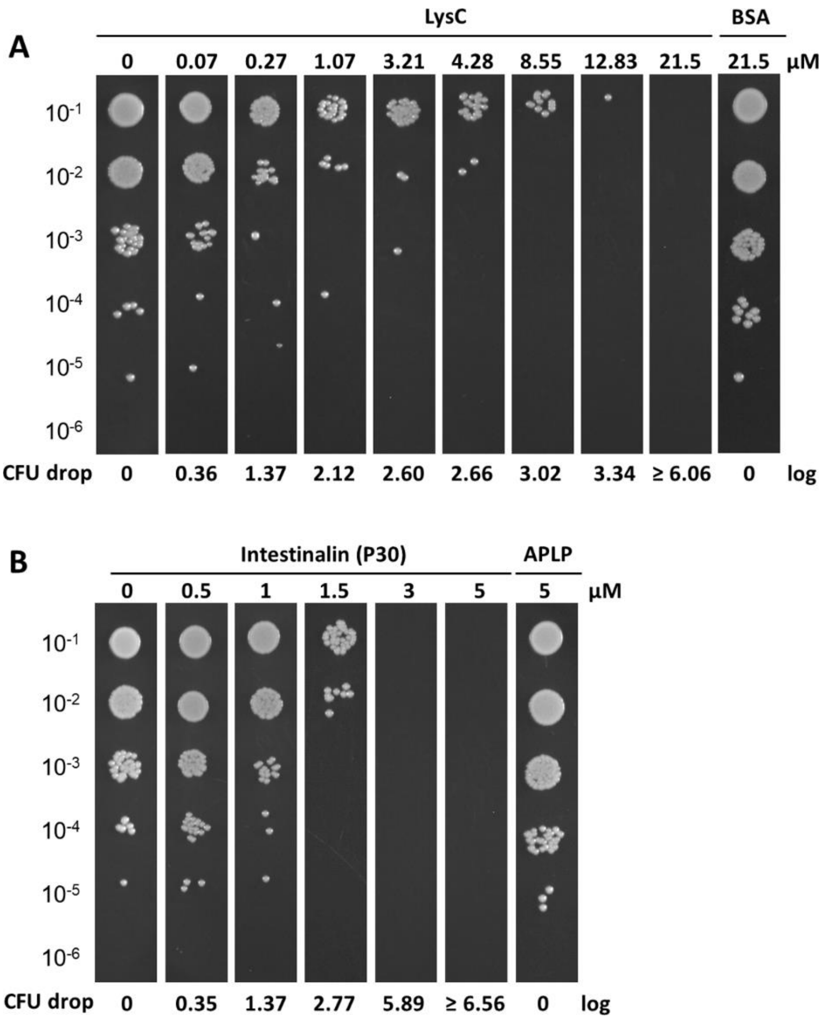

Figure S1. Spot dilution assays showing dose-dependent activity of LysC lytic enzyme and Intestinalin (P30) peptide against *S. aureus* ATCC 25923. Standard antibacterial assays evaluated the activity using (A) LysC at concentrations of 0 – 21.5 µM and (B) Intestinalin at a concentration range of 0 – 5 µM. 5-µl drops containing serial, 10-fold dilutions (from 10^{-1} to 10^{-6}) of reaction mixtures were spotted onto a solid TSB medium. Plates were incubated at 37°C overnight and then photographed. Bovine serum albumin (BSA) at a concentration of 21.5 µM and APLP peptide at a concentration of 5 µM served as negative controls for LysC and Intestinalin (P30) peptide respectively. CFU drops were shown in the logarithmic scale.

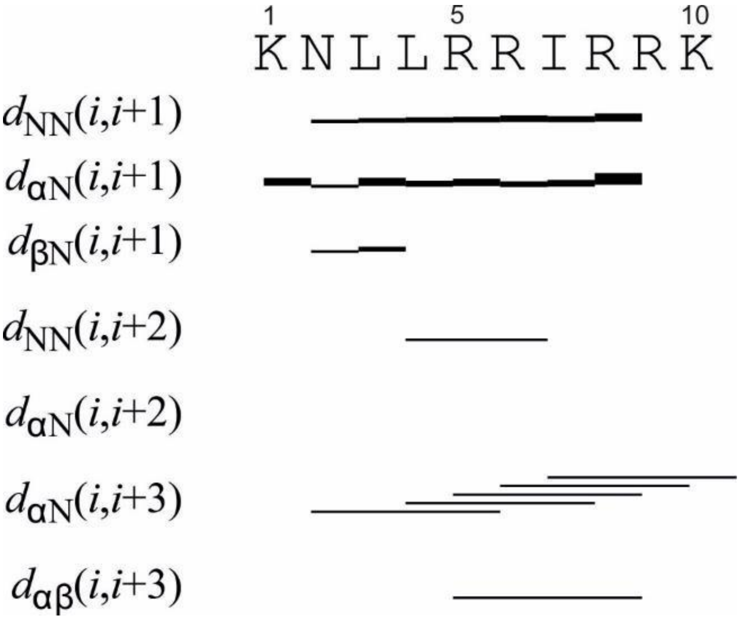

Figure S2. ROE effects correspond to the interproton distances of Intestinalin (P10) at T = 25°C. The bars' thickness reflects the ROE correlation's strength as a strong, medium, or weak.

Table S1. Atoms of residues at separation |i – j| > 1, between which the ROE peaks were found at 25°C for Intestinalin (P10) peptide.

ROE peaks between residues |i – j| > 1 at 25 °C
ε**K**1 – δ**R**6
α**N**2 – H_N**R**5
β**N**2 – β**R**5
β**N**2 – γ**R**5
β**N**2 – δ**R**6
β**N**2 – β**R**5
β**N**2 – γ**R**6
β**N**2 – δ**R**6
δ**N**2 – δ**L**4
α**L**3 – α**I**7
α**L**3 – γ**I**7
β**L**3 – α**I**7
H_N**L**4 – H_N**R**6
H_N**L**4 – β**R**8
α**L**4 – H_N**I**7
α**R**5 – H_N**R**8
α**R**5 – α**R**8
α**R**5 – β**R**8
α**R**6 – H_N**R**9
α**I**7 – α**R**9
α**I**7 – H_N**K**10

Table S2. ^{1}H chemical shifts (ppm) of Pep10_SDS in SDS-d25/D_2O at 25°C.

Residue	Chemical shift [ppm]						
	NH	α-CH	β-CH	γ-CH	δ-CH	ε-CH	ζ-CH<br>η-CH
**Lys 1**	8.61	3.98	1.84	1.40	1.62	2.91	7.34
**Asn 2**	8.60	4.75	$\beta_1$2.79;<br>$\beta_2$2.67		$\delta_1$7.43;<br>$\delta_2$6.76		
**Leu 3**	8.16	3.97	1.67	1.52	$\delta_1$0.83;<br>$\delta_2$0.78		
**Leu 4**	7.97	3.98	1.85	1.64	$\delta_1$0.83;<br>$\delta_2$0.75		
**Arg 5**	7.60	3.93	1.73	1.56	3.07	7.00	η_1/η_2 na
**Arg 6**	7.80	4.02	$\beta_1$1.83;<br>$\beta_2$1.65	1.53	3.07	7.00	η_1/η_2 na
**Ile 7**	7.65	3.78	1.85	γ_{11}1.55;<br>γ_{12}1.08<br>$\gamma_2$0.80	0.75		
**Arg 8**	7.76	4.09	$\beta_1$1.84;<br>$\beta_2$1.66	1.54	3.06	7.00	η_1/η_2 na
**Arg 9**	7.81	4.10	$\beta_1$1.82;<br>$\beta_2$1.73	1.58	3.07	7.00	η_1/η_2 na
**Lys 10**	7.81	4.07	1.75	1.36	1.58	2.89	7.30
**C-NH_2**	$N_1$7.24;<br>$N_2$6.90						

na – not assigned

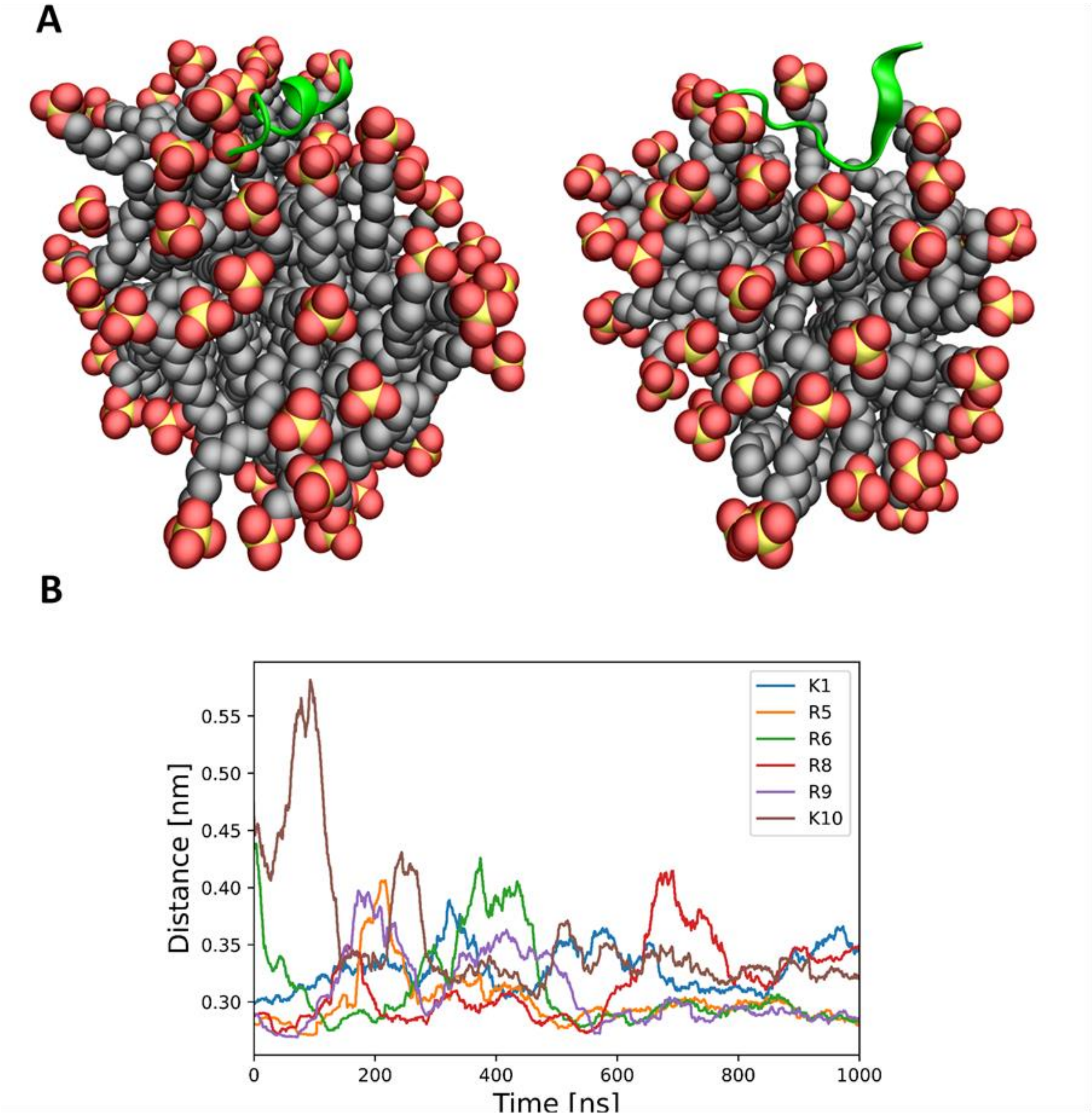

Figure S3. Molecular dynamics simulation of SDS with Intestinalin (P10). (A) Two clusters of P10 from equilibrium simulation: alpha-helical (left, 35% of the population in MD) and random coil (right, 65% of the population in MD). (B) Time evolution of the minimal distances between the side chains of P10 polar residues and the SDS sulfate groups.

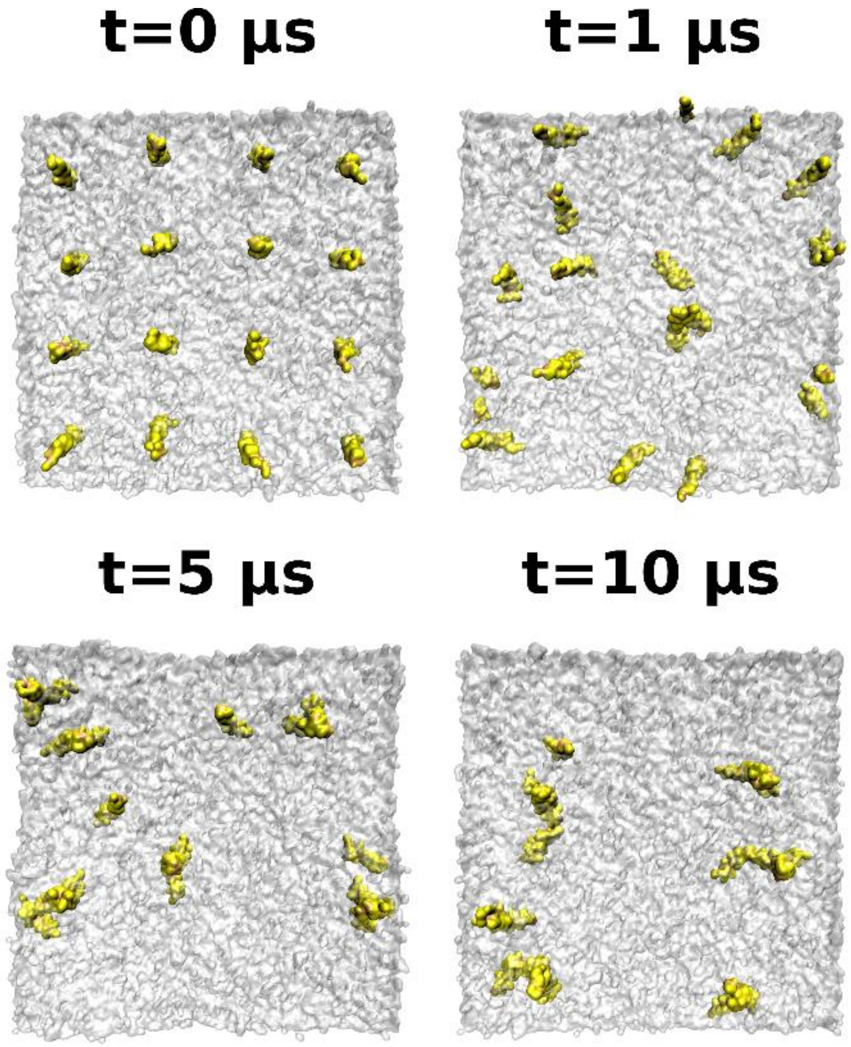

Figure S4. MD simulation snapshots showing the position of Intestinalin (P30) peptides in the membrane at the beginning of the simulation (t = 0 μs) and after the time evolution of the system (at t = 1 μs, 5 μs, or 10 μs). During simulation, all P30 peptides remained in close contact with the membrane and tended to aggregate into small oligomers.

Table S3. Electrostatic interactions for Intestinalin (P30).

A. Electrostatic interactions for P30 dimer [kJ/mol]

	Polar heads of lipids	Water	Helical part of $P30_2$
Helical part of $P30_1$	-22789.7 ±1168.64	-1794.24 ±835.97	2393.06 ±408.40
Helical part of $P30_2$	-23283.5 ±1273.17	-1898.55 ±917.75	-
The energy of lipid head groups rearrangement: -9556,96 kJ/mol			

B. Electrostatic interactions for P30 trimer [kJ/mol]

	Polar heads of lipids	Water	Helical part of $P30_2$	Helical part of $P30_3$
Helical part of $P30_1$	-21585.7 ±910.89	-2227.94 ±806.55	2411.47 ±289.04	2106.37 ±451.20
Helical part of $P30_2$	-19972.5 ±817.58	-2051.26 ±749.39	-	1771.66 ±304.07
Helical part of $P30_3$	-20366.1 ±800.54	-2389.93 ±796.63	-	-
The energy of lipid head groups rearrangement: -17347.20 kJ/mol				

C. Electrostatic interactions for P30 tetramer [kJ/mol]

	Polar heads of lipids	Water	Helical part of $P30_2$	Helical part of $P30_3$	Helical part of $P30_4$
Helical part of $P30_1$	-22593.4 ±915.80	-1747.59 ±754.27	1564.2 ±259.54	1987.97 ±345.54	3156.7 ±421.79
Helical part of $P30_2$	-22789.5 ±1148.65	-1721.74 ±914.14	-	3314.18 ±419.52	355.881 ±118.81
Helical part of $P30_3$	-22748.9 ±1084.00	-2334.06 ±858.05	-	-	1480.71 ±367.66
Helical part of $P30_4$	-23451.7 ±1182.48	-1710.74 ±987.02	-	-	-
The energy of lipid head groups rearrangement: -22796.65 kJ/mol					

Table S4. Simulated systems details.

System	Number of POPG molecules	Number of TLCL2 molecules	Number of water molecules	Number of K+ molecules	Number of Cl- molecules	Approximate box size [Å]
**P30 dimer**	128	32	10244	201	27	8.1 × 8.1 × 9.3
**P30 trimer**	124	31	10306	186	27	8.1 × 8.1 × 9.3
**P30 tetramer**	160	40	13292	239	35	9.2 × 9.2 × 9.3
**P30 pentamer**	192	48	15849	285	42	10 × 10 × 9.3

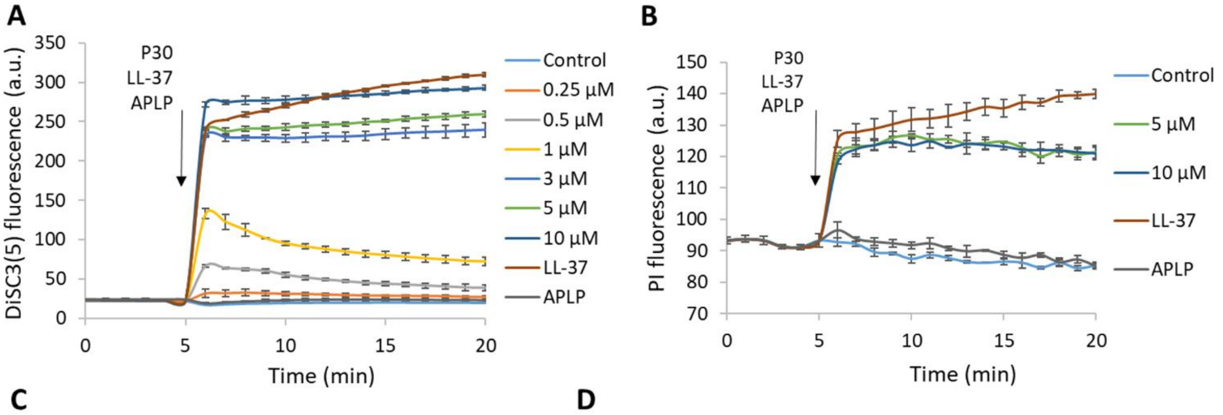

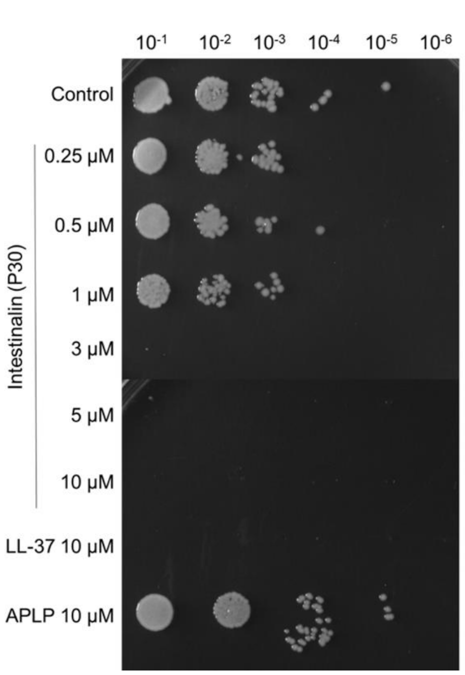

D

Peptide	Concentration (µM)	Reduction level (log CFU/mL)
**P30**	0.25	0.22 ± 0.02
	0.5	0.59 ± 0.03
	1	1.05 ± 0.01
	3	5.00 ± 0.06
	5	6.00 ± 0.06
	10	6.00 ± 0.06
**LL-37**	10	6.00 ± 0.06
**APLP**	10	0.05 ± 0.01

Figure S5. Membrane depolarization (DiSC3(5)), permeabilization (PI), and killing of *S. aureus* ATCC 25923 cells by Intestinalin (P30). Experiments were performed with 10^6 CFU/mL of *S. aureus* ATCC 25923 cells in 20 mM HEPES, pH 7.4 containing 1 µM $DiSC_3(5)$ (λex = 652 nm, λem = 672 nm) and 5 µg/mL PI (λex = 535 nm, λem = 617 nm) at 37°C. The CFU counts were determined at 60 min incubation. 10 µM LL-37 antimicrobial peptide and 10 µM APLP peptide served as a positive and negative control, respectively. (A) The kinetics of DiSC3(5); (B) The kinetics of PI; error bars indicate standard deviation. (↓) peptides addition. (C) Spot dilution assay showing dose-dependent killing activity of Intestinalin (P30) peptide against *S. aureus* ATCC 25923. (D) Reduction levels in log CFU/mL of *S. aureus* ATCC 25923 cells after incubation with Intestinalin (P30) at final concentrations between 0.25 µM to 10 µM; 10 µM LL-37 peptide, and 10 µM APLP peptide.
